# Effects of the informed health choices secondary school intervention after 1 year: a prospective meta-analysis using individual participant data

**DOI:** 10.1186/s13063-024-08577-w

**Published:** 2024-10-30

**Authors:** Faith Chesire, Michael Mugisha, Ronald Ssenyonga, Christopher J. Rose, Allen Nsangi, Margaret Kaseje, Nelson K. Sewankambo, Matt Oxman, Sarah E. Rosenbaum, Jenny Moberg, Astrid Dahlgren, Simon Lewin, Mahima Venkateswaran, Eleni Papadopoulou, Andrew D. Oxman

**Affiliations:** 1 Tropical Institute of Community Health and Development, Kisumu, Kenya; 2 School of Public Health, College of Medicine and Health Sciences, University of Rwanda, Kigali, Rwanda; 3 College of Health Sciences, Makerere University, Kampala, Uganda; 4 Institute of Health and Society, Faculty of Medicine, University of Oslo, Oslo, Norway; 5 Centre for Epidemic Interventions Research, Norwegian Institute of Public Health, Postboks 222 Skøyen, Oslo, 0213 Norway; 6 Faculty of Health Sciences, Oslo Metropolitan University, Oslo, Norway; 7 Department of Health Sciences Ålesund, Faculty of Medicine and Health Sciences, Norwegian University of Science and Technology (NTNU), Ålesund, Norway; 8 Health Systems Research Unit, South African Medical Research Council, Cape Town, South Africa; 9 Global Health Cluster, Norwegian Institute of Public Health, Oslo, Norway

**Keywords:** Adolescents, Critical health literacy, Critical thinking, Health education, Individual participant-level data meta-analysis, Prospective meta-analysis, Secondary school

## Abstract

**Background:**

Critical thinking about health choices is essential to avoid being misled by unreliable information and to use reliable information appropriately. The aim of this prospective meta-analysis was to synthesize the results of 1-year follow-up data from three cluster-randomized trials of an intervention designed to teach lower secondary school students to think critically about health choices. Only one other randomized trial has evaluated a school-based intervention to teach adolescents to think critically about health choices. That trial compared two teaching strategies to teach statistical reasoning. It did not assess long-term learning-retention.

**Methods:**

We conducted the trials in Kenya, Rwanda, and Uganda. The intervention included providing a 2–3-day teacher training workshop and digital resources for ten lessons. The intervention focused on nine key concepts. We did not intervene in control schools. The primary outcome was a passing score on a test (≥ 9 of 18 multiple-choice questions answered correctly). We performed random effects meta-analyses to estimate the overall intervention effects. We calculated learning retention as the test results in the intervention schools after 1 year relative to just after the intervention, adjusted for chance.

**Results:**

Altogether, 244 schools (11,344 students) took part in the three trials. Follow-up data was collected for 8298 students (73%). The overall odds ratio for the primary outcome after 1 year was 3.6 (95% CI: 1.9–7.1; *p* = 0.0001) in favor of the intervention, whereas it was 5.5 (95% CI: 3.0–10.2) just after the intervention. This corresponds to 25.6% (95% CI: 21.1–30.0%) more students in the intervention schools passing the test after 1 year versus 33.3% (95% CI: 28.7–37.8%) just after the intervention. Overall, 2273 (52.6%) of 4324 students in intervention schools had a passing score after 1 year compared to 3397 (58.1%) of 5846 students just after the intervention, indicating 88.3% learning retention.

**Conclusions:**

One year after the intervention, we still found a positive effect on the ability of students to think critically about health choices, but 5.5% fewer students in the intervention schools had a passing score. The certainty of the evidence was also lower due to 27% of students being lost to follow-up.

**Trial registration:**

The protocol for this prospective meta-analysis was registered with PROSPERO May 31, 2022, ID 336580. The three randomized trials were registered in the Pan African Clinical Trial Registry February 15, 2022, PACTR202203880375077; April 5, 2022, PACTR20220488391731; and April 14, 2022, PACTR202204861458660.

**Supplementary Information:**

The online version contains supplementary material available at 10.1186/s13063-024-08577-w.

## Introduction

Being able to understand and apply key concepts for thinking critically about health choices is essential for avoiding being misled by unreliable information and using reliable information appropriately [1]. There have been few rigorous evaluations of interventions to improve adolescents’ understanding and use of such concepts [2, 3].

We used a human-centered design approach [4] to develop the *Be Smart about your Health* digital educational resources [5] for lower-secondary schools in Kenya, Rwanda, and Uganda. The resources included 10 lesson plans with learning goals based on nine key concepts (Box S1) for assessing health claims and making informed health choices [6]. The resources are accessed and used with a web browser on a smartphone or computer. There are two versions of each lesson plan: a version for classrooms with a projector and a version for classrooms with only a blackboard or flipchart.

The 10 lessons were designed to be taught in a single school term. We evaluated the effects of the intervention at the end of the school term in randomized trials in each country [7–9]. The primary outcome was a passing score on *Critical Thinking about Health* test [10, 11]. Altogether, 244 schools (11,344 students) took part in the three trials. The overall adjusted odds ratio across the three countries was 5.5 (95% CI: 3.0–10.2; *p* < 0.0001) in favor of the intervention, which corresponds to 33% (95% CI: 25–40%) more students in the intervention schools passing the test [12].

We measured the same outcomes in all three countries 1 year later. The aim of this study was to synthesize those results. Our objectives were to estimate the intervention effects and assess long-term retention of what was learned, use (transfer) of what was learned, and potential adverse effects.

## Methods

### Study design

This was a prospective meta-analysis. The studies were included, and hypotheses and analysis strategies were specified before any results were known [13]. The three randomized trials that were included were planned collaboratively to ensure a common set of outcome measures and the availability of data to conduct the analyses using individual participant data [14–16]. There were important differences between the three countries (Box S2). At the same time, collaboration on the design of the trials enabled investigation of potential effect modifiers as well as estimating overall effects across the three trials. Details of the methods that were used can be found in the trial protocols, the protocol for this meta-analysis, an addendum to the protocol [17], and reports of the results just after the intervention [7–9, 12].

### Data sources and inclusion criteria

The eligibility criteria for this meta-analysis are shown in Table S1. Because the intervention was not yet available to other investigators, it was not necessary to search for other studies. *Be smart about your Health* has not yet been translated or adapted for use in other countries and there are no registered protocols for evaluating the intervention in other countries.

### Study settings

We conducted context analyses in each country to inform the design of the intervention [18–20]. The contexts are summarized in Box S2. Critical thinking about health was not explicitly included in the standard curriculum and was not being taught in any of the three countries, and teachers did not have resources for teaching critical thinking about health.

### Participant characteristics, random allocation and masking

In each trial, schools were randomly selected and randomly allocated 1:1 to the intervention or a comparison group using block stratified random sequences. Table S2 summarizes the eligibility criteria for schools, teachers, and students and the stratification variables for each of the trials. In all three trials, concealed allocation was conducted by a statistician who was not involved in the recruitment of schools or the analysis of data. No changes were made to the lists after random allocation by the statistician.

Because of the nature of the intervention, it was not possible to blind the research team, teachers, or students. The investigators informed the teachers and students of the purpose of the *Critical Thinking about Health* test used to measure outcomes in both arms of the trial but did not show them the test until the end of the school term when they were asked to complete it. The investigators retrieved and kept all copies of the test, including blank tests, to prevent participants from practicing for the 1-year follow-up.

### Intervention characteristics

**Fig. 1:**
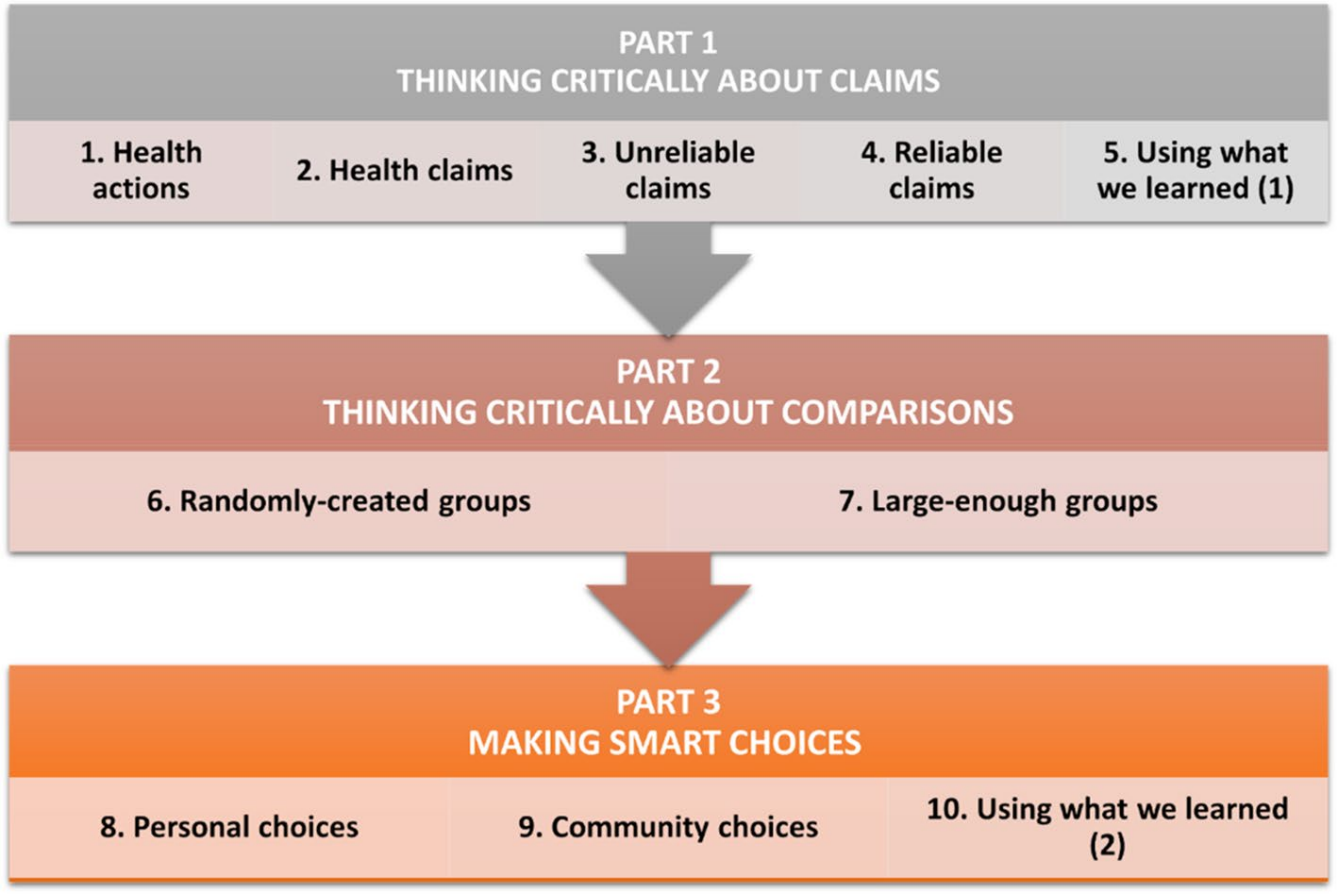
Overview of the 10 *Be Smart about your Health* lessons

Characteristics of the intervention are described using the *Guideline for reporting evidence-based practice educational interventions and teaching* (GREET) checklist (Appendix 1) [21]. The intervention included 2–3-day training workshops for teachers in the intervention schools just prior to the school term during which they taught the IHC lessons. Teachers who participated in pilot testing the resources or were in the national teacher networks that helped to develop the resources facilitated the workshops using presentations included in the *Be Smart about Your Health* resources. The 10 lessons in the resources were designed to each be taught in a single 40-min school period. An overview of the lessons is shown in Fig. 1. The resources also include a teachers’ guide. We did not intervene in the control schools, which followed the standard curriculum in each country.

### Outcomes

The *Critical Thinking about Health* (CTH) test (Appendix 2) [10] was completed by students and teachers in both the intervention and control schools at the end of the school term when the lessons were taught and 1 year later. The test included 18 multiple-choice questions (MCQs), two for each of the nine key concepts included as learning goals. The primary outcome was the proportion of students with a passing score on the CTH test (at least 9 of 18 MCQs answered correctly) [11]. Secondary outcomes are described in Table S3.

For the 1-year follow-up, we included questions about transfer (use of what was learned) and potential adverse effects in the questionnaires completed by students and teachers in the intervention schools (Appendix 3) [17]. We also assessed use of what was learned by asking students in both intervention and control schools to recall a claim about the effects of a health action using a “diary” (Appendix 4) [17]. For each claim, we asked questions to assess their ability to identify and assess the claims and decide what to do. We randomly selected 10 students from each school to complete the diary, and we coded their responses to the questions using the rubric in Appendix 5.

### Statistical analysis

#### Random effects meta-analyses

Country (trial) is a categorical variable but is not exhausted—i.e., there are more levels (countries) than are available in the sample. Therefore, as per the protocol [22], we used random effects meta-analysis to estimate average adjusted odds ratios for students and teachers achieving passing and mastery scores (≥ 9 and ≥ 14 of 18 MCQs answered correctly, respectively) and average adjusted differences in students’ and teachers’ mean scores. We estimated trial-level (aggregate) intervention effects using the same data and analyses as for the original trials. The trial-level estimates for students accounted for the cluster design using random intercepts at the level of school (the unit of randomization). Because there was a one–one relationship between teachers and schools, no such adjustment was necessary for teachers’ outcomes. Odds ratios were meta-analyzed on the log odds ratio metric. Forest plots for the main outcomes include point estimates of between-trial variance (τ^2^), *I*^2^ values to quantify heterogeneity, the results of χ^2^ tests of homogeneity, and, for passing and mastery, estimates of adjusted risk differences.

### Individual participant data (IPD) meta-analyses using generalized linear mixed models

We used generalized linear mixed models to perform IPD meta-analyses, using the original data from the three trials. We estimated adjusted odds ratios for students and teachers achieving passing and mastery scores (logistic regression: logit link, Bernoulli errors) and adjusted differences in students’ and teachers’ mean scores (linear regression: identity link, normal errors), assuming common treatment effects across the trials. We adjusted for school ownership (public or government-funded versus private), use of a projector (versus blackboard), and school performance as fixed effects to account for the stratification used in the original trials as planned. School location (rural vs urban) was a stratification factor for the Kenya trial but not the others, and these data were only collected in the Kenya trial, so we could not adjust for school location as planned [17].

#### Cluster effects, re-expression of odds ratios, handling of missing data, and learning retention

For outcomes measured on students, we used hierarchical random intercepts to account for clustering of students within schools and schools within trials (countries). For outcomes measured on teachers, we used random intercepts to account for clustering of teacher within trial. Because there was a one–one relationship between teachers and schools, there was no clustering of teachers within schools. To aid interpretation, we re-expressed odds ratio estimates as adjusted differences, accounting for uncertainty of the control odds as well as the odds ratios. Test answers that were missing from participants not lost to follow-up were counted as wrong answers. All children and teachers who completed the test were included and analyzed in the arms to which they were randomized.

Retention of what was learned by students and teachers in the intervention schools is reported as the test scores in the intervention schools after 1 year relative to the test scores just after the intervention. We adjusted retention for chance by subtracting the probability of answering questions correctly by chance (guessing) alone from each proportion for passing and mastery scores (10.76% and 0.0145% respectively), from each mean for the mean scores (33.33%), and from the proportion of students answering both questions correctly for each concept (11.11%).

### Addition of interaction terms to the IPD meta-analysis models

We added interaction terms to the IPD meta-analysis models described above to estimate interactions between the intervention and four potential modifiers of the intervention effect for passing, mastery, and score for students: use of a projector (versus blackboard), class size (number of students, a continuous variable), performance on exams at the end of the previous school term (low versus moderate or high), and sex (female versus male). We specified the first three in the protocol with the hypotheses and rationales shown in Table S4. We did not have an a priori hypothesis for sex, which was not specified in the protocol.

To inform judgements about the credibility of the effect modifier analyses, we repeated the analyses of interaction terms for each trial to estimate trial-specific interactions. These analyses were not pre-specified. It was not possible to estimate trial-specific interactions for use of a projector since none of the control schools in Kenya used a projector while all schools in Rwanda used a projector.

We performed prespecified subgroup analyses to estimate intervention effects for passing, mastery, and score in students lacking English reading proficiency. English reading proficiency was assessed using four questions at the beginning of the CTH test: two advanced and two basic questions. We categorized students’ reading skills as “advanced” (all four questions correct), “basic” (both basic questions correct and one or both advanced questions wrong), and “lacking basic” (one or both basic questions wrong).

#### Additional questions about transfer and adverse effects

For each additional question about transfer and adverse effects, we report the number and proportion of participants who selected each response option. For questions with three or more responses options, we also dichotomized the responses (e.g., “stressful or very stressful” vs “not at all stressful or a little stressful”).

#### Confidence intervals and p-values

We report two-sided 95% confidence intervals and two-sided *p*-values, where appropriate, throughout. All statistical analyses were performed using Stata 18 (StataCorp LLC, College Station, Texas, USA).

#### Sensitivity analyses

We conducted two pre-specified sensitivity analyses to explore the risk of bias due to attrition. First, we did a weighted analysis using inverse probability weighting. Second, we calculated upper and lower bounds for the mean difference in test scores using the Lee bounds approach [23].

### Risk of bias and certainty assessment

Two researchers (see Acknowledgements) independently assessed the risk of bias for passing, mastery, and mean scores for students and teachers in each trial using the revised Cochrane Collaboration’s tool for assessing risk of bias in cluster-randomized trials [24]. They were not involved in the design, implementation, interpretation, or reporting of the three trials or this meta-analysis. The same two researchers assessed the certainty of the evidence for each outcome using the Grading of Recommendations Assessment, Development, and Evaluation (GRADE) system [25, 26]. The authors assessed the credibility of subgroup differences (potential effect modifiers) using the Instrument to assess the Credibility of Effect Modification Analyses (ICEMAN) [27].

## Results

The three trials, conducted during the same academic year, between April and August 2022, included a total of 244 schools with one teacher at each school (Table 1). All the teachers and 11,344 students completed the CTH test the first time it was administered (5431 in the control schools and 5913 in the intervention schools). The test was administered again in all 244 schools 1 year later. This time, 104 teachers in the control schools (85.2%) and 108 in the intervention schools (88.5%) completed the test. In the control schools, 3942 (72.1%) of the students completed the test, and 4356 (73.5%) of the students in the intervention schools completed the test after 1 year.

**Table 1:** Participant characteristics at 1-year follow-up

		**Control schools** *N* (%)	**Intervention schools** *N* (%)
**Schools**		122	122
Country	Kenya	40 (32.8%)	40 (32.8%)
	Rwanda	42 (34.4%)	42 (34.4%)
	Uganda	40 (32.8%)	40 (32.8%)
Ownership	Public or government-aided	89 (73.0%)	92 (75.4%)
	Private	33 (27.0%)	30 (24.6%)
**Teachers**		122	122
Completed tests		104 (85.2%)	108 (88.5%)
Country^a^	Kenya	34 (27.9%)	33 (27.0%)
	Rwanda	35 (28.7%)	35 (28.7%)
	Uganda	35 (28.7%)	40 (32.8%)
Education^a^	Diploma	30 (24.6%)	22 (18.0%)
	Bachelor’s degree	71 (58.2%)	84 (68.9%)
	Master’s degree	3 (2.5%)	1 (0.8%)
	Missing	0 (0.0%)	1 (0.8%)
Years of experience^ab^	Median (IQR)	9 (4 to 13)	9 (3 to 11)
Sex^ac^	Female	21 (17.2%)	25 (20.5%)
	Male	48 (39.3%)	48 (39.3%)
**Students**		5466	5927
Completed tests		3942 (72.1%)	4356 (73.5%)
Country^a^	Kenya	1077 (27.3%)	1369 (31.4%)
	Rwanda	1181 (30.0%)	1238 (28.4%)
	Uganda	1684 (42.7%)	1749 (40.2%)
Completed tests per class	Median (IQR)	48 (38 to 63)	51 (41 to 69)
Sex^a^	Female	2282 (57.9%)	2266 (52.0%)
	Male	1659 (42.1%)	2089 (48.0%)
Age (years) at time of intervention^a^	Mean (SD)	15.7 (1.2)	15.6 (1.1)

^a^Data are for participants who took the test

^b^Data for the Rwanda and Uganda trials only

^c^Data for the Kenya and Uganda trials only

For students who completed the 1-year follow-up test, the mean age at time of intervention was 15.7 years (SD 1.2) in the control schools and 15.6 years (SD 1.1) in the intervention schools, while for all students, the mean age at time of intervention was 15.8 (SD 1.2) in control schools and 15.7 (SD 1.1) in intervention schools. For students who completed the 1-year follow-up test, the proportion of girls was 57.9% in the control schools and 52.0% in the intervention schools, compared to 56.6% and 51.7% respectively for all the students at time of intervention.

### Primary outcome

Based on the IPD meta-analysis, the overall odds ratio for the primary outcome—the proportion of students with a passing score (≥ 9 of 18 correct answers)—after 1 year was 3.7 (95% CI: 2.9–4.6; *p* < 0.0001) in favor of the intervention (Table 2) compared to 5.7 (4.5–7.4) just after the intervention. This corresponds to 25.6% (95% CI: 21.1–30.0%) more students in the intervention schools passing the test after 1 year, compared to 33.3% (95% CI: 28.7–37.8%) just after the intervention.

**Table 2:** Main results—individual participant data meta-analyses

	**Control schools**	**Intervention schools**	**Adjusted difference**	**Odds ratio**	***p***	**ICC**	
	3942 students^a^ 122 schools	4356 students^a^ 122 schools				**Trial**	**School**
**Primary outcome**							
Students with a passing score (≥ 9/18)	1123/3918 (28.7%)	2273/4324 (52.6%)	25.6% (21.1–30.0)	3.7 (2.9–4.6)	< 0.0001	0.005	0.159
**Secondary outcomes**							
**Students**							
Students with a mastery score (≥ 14/18)	93/3918 (2.4%)	672/4324 (15.5%)	12.4% (9.9–14.8)	8.1 (5.6–11.8)	< 0.0001	< 0.001	0.227
Mean score for students^b^	38.8% (16.8)	50.7% (21.5)	12.3% (10.2–14.4)		< 0.0001	< 0.001	0.166
**Teachers**							
Teachers with a passing score (≥ 9/18)	64/104 (61.5%)	101/108 (93.5%)	31.8% (15.3–48.3)	10.9 (4.4–27.2)	< 0.0001	0.165	
Teachers with a mastery score (≥ 14/18)	13/104 (12.5%)	79/108 (73.1%)	60.9% (47.5–74.2)	24.7 (11.0–55.2)	< 0.0001	0.116	
Mean score for teachers^b^	54.0% (18.9)	80.7% (15.8)	27.0% (22.6–31.3)		< 0.0001	0.113	

Data are *n*/*N* (%), % (95% CI), or % (SD). Clustering was accounted for using random intercepts at the level of trial and, for outcomes measured on students, random intercepts at the level of school within trial. Logistic regression was used to estimate adjusted odds ratios for passing and mastery, which are re-expressed as adjusted differences. Linear regression was used to estimate adjusted differences in mean scores. Intraclass correlation coefficients (ICCs) are estimated at the same levels as the random intercepts (trial and school within trial). Fixed effects were used to adjust all estimates as described in the Methods section. Wald-type confidence intervals and two-sided *p*-values were computed in all analyses

^a^24 students in control schools and 32 students in intervention schools were not included in the analyses because of incomplete performance data. The analyses are adjusted for performance, so complete data on this variable is required

^b^Average percent correct answers

Overall, 52.6% of students in intervention schools had a passing score after 1 year compared to 58.1% just after the intervention (88.3% learning retention) (Table 3).

**Table 3:** Retention of what was learned in intervention schools

		Control*	Intervention*	Adjusted difference*	**Retention**^**♱**^
**Students**					
**Passing score** (≥ 9/18)	**Initial**^**a**^	1385/5388 (25.7%)	3397/5846 **(58.1%)**	33.3% (28.7–37.8)	**88.3%**
	**One year**	1123/3918 (28.7%)	2273/4324 **(52.6%)**	25.6% (21.1–30.0)	
**Mastery score** (≥ 14/18)	**Initial**^**a**^	76/5388 (1.4%)	1149/5846 **(19.7%)**	18.1% (15.1–21.0)	**77.4%**
	**One year**	93/3918 (2.4%)	672/4324 **(15.5%)**	12.4% (9.9–14.8)	
**Mean score**	**Initial**^**a**^	36.7%	**54.1%**	17.5% (15.3–19.7)	**83.6%**
	**One year**	38.8%	**50.7%**	12.3% (10.2–14.4)	
**Teachers**					
**Passing score** (≥ 9/18)	**Initial**^**a**^	80/122 (65.6%)	118/122 **(96.7%)**	32.7% (23.8–41.6)	**96.3%**
	**One year**	64/104 (61.5%)	101/108 **(93.5%)**	31.8% (15.3–48.3)	
**Mastery score** (≥ 14/18)	**Initial**^**a**^	12/122 (9.8%)	102/122 **(83.6%)**	74.9% (66.7–83.1)	**87.3%**
	**One year**	93/3918 (2.4%)	672/4324 **(15.5%)**	60.9% (47.5–74.2)	
**Mean score**	**Initial**^**a**^	53.5%	**85.4%**	32.5% (28.5–36.4)	**91.0%**
	**One year**	38.8%	**50.7%**	27.0% (22.6–31.3)	

^a^Just after the intervention

^*^Based on data from the individual participant data meta-analysis (Table S5). Twenty-four students in control schools and 32 students in intervention schools were not included in the analyses because of incomplete performance data. The analyses are adjusted for performance, so complete data on this variable is required

^†^Retention of what was learned by students and teachers in the intervention schools reported as the test scores in the intervention schools after 1 year relative to the test scores just after the intervention. Retention is adjusted for chance by subtracting the probability of answering questions correctly by chance (guessing) alone from proportions of passing and mastery scores and the mean scores (10.76%, 0.0145%, and 33.33% respectively)

Like the results when the test was first administered, the odds ratios are heterogeneous across the three trials (*I*^2^ = 90.2%, χ^2^ = 14.1, *p* = 0.0008) (Fig. 1), and the adjusted differences are more homogeneous. This is because the smaller odds ratios corresponded to larger proportions of students with a passing score in the control schools. As before, both the odds ratio and the adjusted difference was larger in Rwanda than in Kenya and Uganda.

**Fig. 2:**
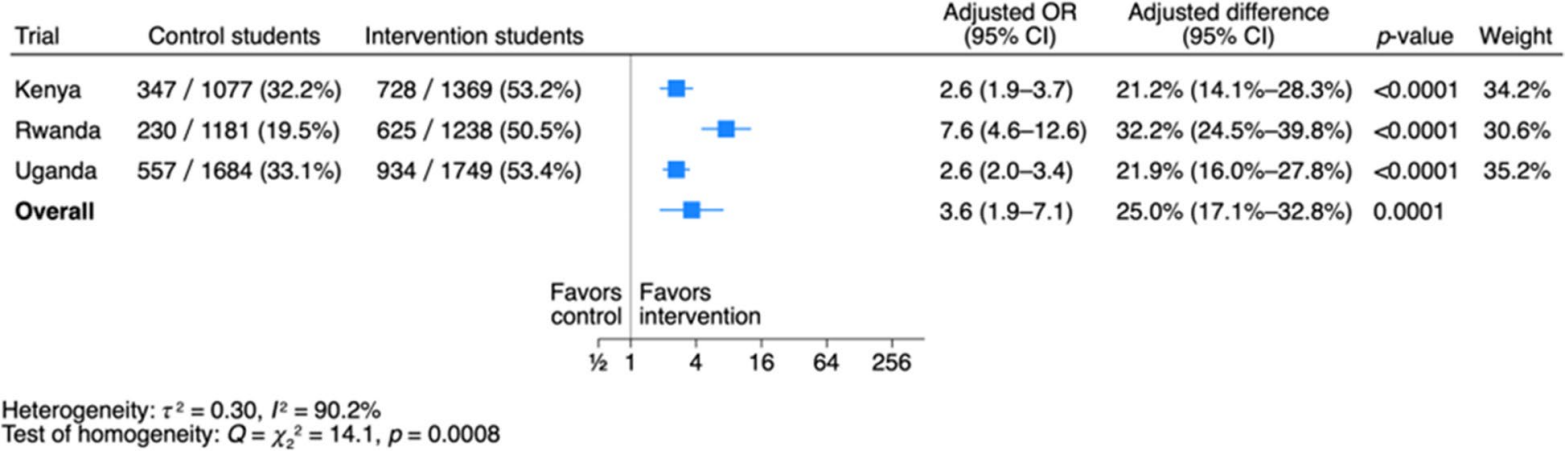
Students with a passing score (≥ 9 out of 18) at 1-year follow-up. Note: This is an inverse variance-weighted random effects meta-analysis using trial-level (aggregate) intervention effects (using the same data and analyses as used in each trial), whereas the meta-analyses in Table 2 using individual participant data are fixed effect meta-analyses

The fixed-effect estimate of the overall odds ratios from the fixed effect IPD meta-analysis (Table 2), the fixed effect IPD meta-analysis using inverse probability weighting (Table S5), and the inverse variance-weighted random effects meta-analysis using aggregated data from each trial (Fig. 2) are similar—3.7 (95% CI 2.9–4.6), 3.7 (95% CI 1.8–7.7), and 3.6 (95% CI 1.9–7.1) respectively.

### Secondary outcomes for students

At 1-year follow-up, the overall adjusted odds ratio for the proportion of students with a score indicating mastery of the key concepts (≥ 14 of 18 correct answers) was 8.1 (95% CI 5.6–11.78) (Table 2) compared to 22.9 (95% CI 14.8–35.4) just after the intervention. This corresponds to an adjusted difference of 12.4% (95% CI 9.9–14.8%) more students mastering the prioritized key concepts after 1 year compared to 18.1% (95% CI 15.1–21.0%) just after the intervention. Unlike the passing scores, the odds ratios for mastery across the three trials are homogeneous (*I*^2^ = 0.0%, χ^2^ = 1.4, *p* = 0.4992) (Fig. S1). The adjusted differences were also similar. The results of the sensitivity analysis using IPW were similar to the results without IPW (Table S5).

Overall, after 1 year, 675 (15.5%) of 4356 students in the intervention schools had a score indicating mastery compared to 19.7% just after the intervention (77.4% learning retention) (Table 3).

At 1-year follow-up, the overall adjusted difference in the mean score (percent correct answers) on the CHT test was 12.3% (95% CI 10.2–14.4%) (Table 2) compared to 17.5% (95% CI 15.3–19.7%) just after the intervention. The adjusted differences for the three trials are heterogeneous (*I*^2^ = 73.3%, χ^2^ = 6.9, *p* = 0.0322) but consistently in favor of the intervention schools, ranging from 9.1% in Kenya to 16.0% in Rwanda (Fig. S2). The results of the sensitivity analysis using IPW were similar to the results without IPW (Table S5).

Loss to follow-up was slightly larger in the control schools (27.9%) compared to the intervention schools (26.5%). The Lee bounds analysis resulted in a lower (worst case) and upper (best case) mean difference of 11.1% and 12.5%, respectively (95% CI 4.4–18.8%). This indicates that with the worst-case scenario, the average test score in the intervention schools was 11.1% higher than in the control schools (with a lower confidence limit of 4.4%).

After 1 year, the mean scores for students in the intervention schools were similar (weighted average 50.7%), compared to 54.1% just after the intervention (learning retention 83.6%) (Table 3).

**Fig. 3:**
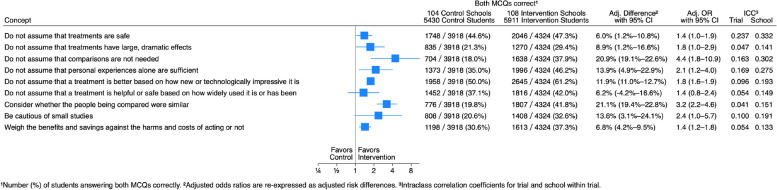
Results for each key concept at 1-year follow-up

Students in the intervention schools answered both questions correctly more often than students in control schools for all nine key concepts (Fig. 3). However, both the relative and absolute effects of the intervention on the ability to apply each of the nine key concepts varied. The odds ratios varied from 1.4 to 4.4, and the adjusted difference varied from 6.0% (for the IHC Key Concept “Do not assume that treatments are safe.”) to 21.1% (for the concept “Consider whether the people being compared were similar.”).

The proportion of students in intervention schools who answered both questions correctly varied from 29.4% (for the concept “Do not assume that treatments have large, dramatic effects.”) to 61.2% (for the concept “Do not assume that a treatment is effective based on how new or technologically impressive it is.”). Learning retention was between 86 and 96% for eight of the nine concepts (Table 4). It was only about 50% for the concept “Do not assume that treatments have large, dramatic effects.”

**Table 4:** Retention of what was learned in intervention schools

Concept	Initial^a^	One-year	Retention^b^
1. Do not assume that treatments are safe	2938/5846 (50.3%)	2046/4324 (47.3%)	92.3%
2. Do not assume that treatments have large, dramatic effects	1847/5846 (31.6%)	1270/4324 (21.3%)	49.7%
3. Do not assume that comparisons are not needed	2493/5846 (41.1%)	1638/4324 (37.9%)	89.3%
4. Do not assume that personal experiences alone are sufficient	2854/5846 (48.8%)	1373/4324 (46.2%)	93.1%
5. Do not assume that a treatment is better based on how new or technologically impressive it is	3706/5846 (63.4%)	2645/4324 (61.2%)	95.8%
6. Do not assume that a treatment is helpful or safe based on how widely used it is or has been	2649/5846 (45.3%)	1816/4324 (42.0%)	90.3%
7. Consider whether the people being compared were similar	2573/5846 (44.0%)	1807/4324 (41.8%)	93.3%
8. Be cautious of small studies	2067/5846 (35.4%)	1408/4324 (32.6%)	88.5%
9. Weigh the benefits and savings against the harms and costs of acting or not	2427/5846 (41.5%)	1613/4324 (37.3%)	86.2%

^a^Just after the intervention

^b^Retention of what was learned by students in the intervention schools reported as the test scores in the intervention schools after 1 year relative to the test scores just after the intervention, adjusted for chance (guessing) by subtracting the probability of answering both questions correctly (11.11%) from each proportion

### Results for teachers

At 1-year follow-up, the overall odds ratio for teachers with a passing score was 10.9 (95% CI 4.4–27.2) (Table 2), compared to 19.1 (95% CI 6.4–56.6) just after the intervention. This corresponds to an adjusted difference of 31.8% (95% CI 15.3–48.3%) more teachers having a passing score, compared to 32.7% (95% CI 23.8–41.6%) just after the intervention. The odds ratios are homogeneous across the three trials (*I*^2^ = 0.0%, χ^2^ = 0.1, *p* = 0.9334) (Fig. S3). Overall, 93.5% of teachers in the intervention schools had a passing score after 1 year, compared to 96.7% just after the intervention (learning retention 96.3%) (Table 3).

The overall odds ratio for teachers with a mastery score was 24.7 (95% CI 11.0–55.2) (Table 2), compared to 61.3 (95% CI 25.6–146.8) just after the intervention. This corresponds to 60.9% (95% CI 47.5–74.2%) more teachers having a mastery score, compared to 74.9% (95% CI 66.7–83.1%) just after the intervention. The odds ratios are homogeneous across the three trials (*I*^2^ = 0.0%, χ^2^ = 1.5, *p* = 0.4709) (Fig. S4). Overall, 79 out of 108 (73.1%) of the teachers in the intervention schools had a mastery score, compared to 83.6% just after the intervention (learning retention 87.3%) (Table 3).

The overall adjusted difference in the mean score for teachers was 27.0% (95% CI 22.6–31.3%) (Table 2), compared to 32.5% (95% CI 28.5–36.4%) just after the intervention. The mean scores varied from 21.5% in Kenya to 31.9% in Rwanda (*I*^2^ = 50.7%, χ^2^ = 4.1, *p* = 0.1315) (Fig. S5). The mean score for teachers in intervention schools was 80.7% compared to 85.4% just after the intervention (learning retention 91.0%) (Table 3).

As for students, loss to follow-up for teachers was slightly larger in the control schools (14.8%) compared to the intervention schools (11.5%). The Lee bounds analysis (Table S5) resulted in a lower (worst case) and upper (best case) mean difference of 26.0% and 28.5%, respectively (95% CI 21.1–34.2%). This indicates that with the worst-case scenario, the average test score in the intervention schools was 26% higher than in the control schools (with a lower confidence limit of 21%).

### Intended behaviors and self-efficacy

There were only minor differences between self-reported intentions after 1 year compared to just after the intervention (Table S6). Students in the intervention schools were more likely than students in control schools to say they would find out if a treatment claim was based on a research study comparing treatments at the 1-year follow-up (OR 1.5, 95% CI 1.2–1.9) and just after the intervention (OR 1.5, 95% CI 1.4–1.7). The adjusted difference in the proportion who responded likely or very likely was 10.1% (95% CI 5.2–15.0%) compared to 10.7% (95% CI 8.3–13.0%) just after the intervention. There was little difference between students in the intervention and control schools in how likely they said they would be to find out what the claim was based on or to say “yes” if invited to participate in a controlled trial.

Students in intervention schools were more likely than students in control schools to respond that they found it easy or very easy to know if a claim about a treatment is based on a controlled trial (OR 1.8, 95% CI 1.3–2.3; adjusted difference 13.0%, 95% CI 6.5–19.5%) (Table S7). They also were more likely to respond that they found it easy or very easy to judge the trustworthiness of the results of a controlled trial (OR 1.4, 95% CI 1.1–1.7; adjusted difference 6.9%, 95% CI 2.0–11.8%) but less so compared to just after the intervention (OR 1.8, 95% CI 1.5–2.1; adjusted difference 12.0%, 95% CI 8.7–15.2%). The odds ratio and adjusted difference between how easy students in intervention compared to control schools found it to assess the relevance of a research study were slightly larger after 1 year compared to just after the intervention (OR 1.4 (95% CI 1.2–1.6); adjusted difference 8.0% (95% CI 4.9–11.0%) compared to OR 1.2 (95% CI 1.1–1.3); adjusted difference 4.9% (95% CI 2.5–7.3%)). There were smaller differences in how easy they thought it is to find information about treatments that is based on controlled trials.

### Transfer and adverse effects

Most (91%) of the students in intervention schools reported that what they learned from the *Be Smart about your Health* lessons was helpful or very helpful (Table S9a), 64% found it more helpful than what they learned in other subjects (Table S9b), and 88% reported that they had used what they learned from the lessons a little or a lot (Table S9c). On the other hand, 10% of the students reported that what they learned from the lessons was less helpful than what they learned in other subjects, and 11% reported that they had not used what they learned.

Students in the intervention schools were more likely to correctly assess the reliability of a claim about the effects of a health action that they encountered during the week prior to when they completed the CTH test (adjusted difference 16.1% (95% CI 8.5–13.7%)) (Table 5). We did not detect substantial differences in the reasons that students gave for deciding whether to take the health action in the claim or advise someone else to take that health action (Table 6).

**Table 5:** Identification and assessment of claims about the effects of health actions

Outcome	Control schools	Intervention schools	Adjusted difference	Odds ratio^a^	*p*	ICC^b^	
	*N* (%)	*N* (%)				Trial^c^	School
Health action correctly identified	781/1251 (62.4%)	873/1234 (70.8%)	8.4% (2.8 to 13.9)	1.6 (1.2 to 2.1)	0.003	0.03	0.22
Claimed effect correctly identified	725/1249 (58.1%)	781/1232 (63.4%)	5.6% (− 0.2 to 11.4)	1.3 (1.0 to 1.8)	0.060	0.03	0.22
Basis for the claim correctly identified^d^	370/697 (53.1%)	462/754 (61.3%)	7.9% (1.0 to 14.8)	1.5 (1.0 to 2.0)	0.025	0.01	0.20
Reliability of the claim correctly assessed^d^	155/371 (41.8%)	285/463 (61.6%)	16.1% (8.5 to 23.7)	2.3 (1.6 to 3.2)	< 0.001	0.22	0.27

10 randomly selected students from each school were asked to identify and assess a claim about the effects of a health action that they had encountered during the past week

^a^Clustering was accounted for using random intercepts at the level of trial and, for outcomes measured on students, random intercepts at the level of school within trial. Logistic regression was used to estimate adjusted odds ratios for passing and mastery, which are re-expressed as adjusted differences

^b^Intraclass correlation coefficients (ICCs) are estimated at the same levels as the random intercepts (trial and school within trial)

^c^The results for each country are shown in Table S10a–S10d in the supporting information

^d^If the health action and claimed effect were not identified correctly, students were not included in the analysis of whether the basis was correctly identified or any of the subsequent assessments. If the basis for the claim was not correctly identified, students were not included in the analysis of whether the reliability of the claim was assessed correctly or any of the subsequent analyses in Table 6

**Table 6:** Reasons for deciding whether to take the health action

Outcome	Control schools	Intervention schools	Adjusted difference	Odds ratio^a^	*p*	ICC^b^	
	*N* (%)	*N* (%)				Trial	School
Reliability of the claim	214/364 (34.1%)	194/460 (42.2%)	6.5% (− 1.3 to 14.4)	1.4 (0.9 to 2.2)	0.100	0.14	0.30
Advantages of the health action	136/359 (37.9%)	144/430 (33.5%)	− 5.4% (− 12.5 to 1.6)	0.7 (0.5 to 1.1)	0.117	0.33	0.44
Disadvantages of the health action	110/360 (30.6%)	168/446 (37.7%)	7.3% (− 0.5 to 15.1)	1.6 (1.0 to 2.6)	0.060	0.17	0.38
Personal experience	265/293 (90.4%)	279/334 (83.5%)	− 6.9% (− 14.3 to 0.7)	0.6 (0.3 to 1.0)	0.056	0.12	0.26
Health professional or researcher advice	25/293 (8.5%)	48/334 (14.4%)	5.8% (− 1.6 to 13.3)	1.7 (0.9 to 3.3)	0.101	0.15	0.32

10 randomly selected students from each school were asked to identify and assess a claim about the effects of a health action that they had encountered during the past week, to say whether they would take the health action, and to say why. The results for each country are shown in Table S10e–S10i in the Supporting information

^a^Clustering was accounted for using random intercepts at the level of trial and, for outcomes measured on students, random intercepts at the level of school within trial. Logistic regression was used to estimate adjusted odds ratios for passing and mastery, which are re-expressed as adjusted differences

^b^Intraclass correlation coefficients (ICCs) are estimated at the same levels as the random intercepts (trial and school within trial)

Most of the students in intervention schools reported challenging their teachers (77.3%), their parents and other adults at home (82.1%), and students or friends (85.4%) based on what they learned in the lessons (Tables S9d to S9f). Challenging their teachers, parents and other adults, and students or friends was experienced negatively (bad or very bad) by 14.0%, 19.8%, and 14.8% of the students, in Kenya, Rwanda, and Uganda, respectively. Some (17.7%) of the students found the lessons stressful or very stressful (Table S9g), and 26.2% reported that the lessons had bad effects or disadvantages for them apart from stress or feeling bad about challenging what others said.

Most (88.1%) of the teachers in intervention schools observed their students using what they learned from *the Be Smart about your Health* lessons sometimes or a lot, whereas 11.0% reported observing their students using what they learned rarely or not at all (Table S11a). Most 91.7%) of the teachers also reported using what they learned from the lessons sometimes or a lot (Table S11b). Most (90.8%) also reported using what they learned from preparing and teaching the lessons for other subjects (Table S11c).

Most (84.4%) of the teachers reported that their students challenged things that they (the teachers) said because of the lessons (Table S11d). This was experienced negatively (bad or very bad) by 10.1% of the teachers. Some (11.0%) of the teachers reported that preparing and teaching the lessons was stressful (Table S11e). When asked what made the lessons stressful, the most frequent responses were completing the lessons during the time available (29.4%) and taking time away from other subjects (24.8%) (Table S11f).

When asked if they observed any of their students relying on any of the unreliable claims that were used as examples in the lessons, 49.5% of the teachers responded that they had observed this sometimes or a lot.

### Effect modifiers

The results for effect modifiers for the primary outcome (passing scores) were similar to the results just after the intervention (Table S12a). However, the tests for interaction do not suggest that chance is an unlikely explanation of the projector version of the lessons appearing to be more effective than the blackboard version, whereas just after the intervention they did. Therefore, the credibility of the projector version being more effective than the blackboard version for the 1-year follow-up results was very low (Table S13), compared to low just after the intervention. Nonetheless, the odds ratios—1.79 (95% CI 0.59 to 5.45, *p* = 0.303 and 1.71 (95% CI 1.02 to 2.86, *p* = 0.040)—were similar.

For the same reason, the credibility of the intervention being more effective for students who performed high or moderate on exams at the end of the previous school term than for low performing students was low for the 1-year follow-up results (OR 1.22; 95% CI 0.95 to 1.57, *p* = 0.126) compared to moderate for the results just after the intervention (OR 1.57; 95% CI 1.15 to 2.16, *p* = 0.005).

In contrast, chance was a less likely explanation of the intervention appearing to be more effective in smaller classes for the 1-year follow-up results (OR for the interaction 0.99; 95% CI 0.98–0.99, *p* = 0.002) compared to the results just after the intervention (OR for the interaction 0.99; 95% CI 0.98–1.00, *p* = 0.102). Consequently, the credibility of the intervention being more effective in smaller classes was high for the 1-year results compared to moderate for the results just after the intervention.

The credibility of the intervention being more effective for boys than for girls was moderate for both the 1-year results (OR 1.33; 1.13 to 1.57, *p* = 0.001) and the initial results (OR 1.49; 1.23 to 1.81, *p* < 0.0001).

Compared to students with advanced English reading proficiency, students who lacked proficiency or had basic proficiency were less likely to have a passing score on the CTH test both just after the intervention and 1 year later (Table S14a).

### Risk of bias and certainty assessment

Just after the intervention, two independent researchers assessed the certainty of the evidence for passing, mastery, and mean scores for students as high. We have downgraded this to moderate due to missing outcome data. Overall, 27% of the students and 13% of teachers enrolled in the trial did not complete the CTH test after 1 year. Although our sensitivity analyses suggest that this is unlikely to have substantially biased the results, we cannot rule out some degree of bias. The risk of bias was therefore assessed as moderate for all the main outcomes in the three trials (Table S15).

The two independent researchers also assessed the certainty of the evidence for the main outcomes. They assessed the certainty of the evidence for all three outcomes for students just after the intervention as high. They downgraded the certainty of the evidence for student outcomes to moderate due to missing outcome data. In addition to the risk of bias, there are wider confidence intervals because of the missing data. This does not affect the certainty of the evidence for mastery and mean scores for students, which were assessed in relation to no effect. However, it could potentially affect the certainty of evidence for the primary outcome, which was assessed in relation to a threshold of 20% (the smallest important difference specified in the trial protocols) [14–16]. Based on the results of the individual participant data (IPD) analysis (shown in Table 4), that confidence interval (21.1%–30.0%) does not cross the 20% threshold. However, based on the results of the random effects meta-analysis (Fig. 2), the confidence interval (17.1% to 32.8%) does cross that threshold. Since both the risk of bias and the wider confidence interval for the primary outcome are due to missing data, they did not downgrade the certainty of evidence twice (both for risk of bias and imprecision). They therefore assessed the certainty of the evidence for all the main outcomes for students as moderate (Table S16).

The two independent researchers assessed all three outcomes for teachers as moderate because of serious imprecision due to insufficient sample size. They did not further downgrade the certainty of evidence from moderate to low because of missing outcome data for teachers. Although this may have resulted in some degree of bias, it appears unlikely that the results were substantially biased by attrition. Based on the Lee bounds, even with the worst-case assumption, the average test score in the intervention schools was 26% higher than in the control schools. In addition, while attrition resulted in wider confidence intervals, even the lower limits of the confidence intervals suggest a large effect for all three outcomes.

The main findings are summarized in Table 7. After 1 year, the IHC secondary school intervention probably increases the proportion of students and teachers with passing and mastery scores, and the mean difference in scores for students and teachers.

**Table 7:** Summary of findings

**Outcomes**^**a**^	**Control schools**^**b**^	**Intervention schools**^**c**^** (95% CI)**	**Relative effect** odds ratio (95% CI)	**Number of participants** (effective sample size)^d^	**Certainty of the evidence** (GRADE)^e^
**Students**					
Passing	28.7%	54.3% (49.8–58.7)	3.7 (2.9–4.6)	8298 (1324)	⊕ ⊕ ⊕ ◯ Moderate certainty^f^
Mastery	2.4%	14.8% (12.3–17.2)	8.1 (5.6–11.8)	8298 (974)	⊕ ⊕ ⊕ ◯ Moderate certainty^f^
Mean score	38.8%	51.1% (49.0–53.2)		8298 (1276)	⊕ ⊕ ⊕ ◯ Moderate certainty^f^
**Teachers**					
Passing	61.5%	93.3% (76.8–100)	10.9 (4.4–27.42	212	⊕ ⊕ ⊕ ◯ Moderate certainty^g^
Mastery	12.5%	73.4% (60.0–100)	24.7 (11.0–55.2)	212	⊕ ⊕ ⊕ ◯ Moderate certainty^g^
Mean score	54.0%	81.0% (76.6–85.3)		212	⊕ ⊕ ⊕ ◯ Moderate certainty^g^

^a^Passing: ≥ 9 of 18 correct answers. Mastery: ≥ 14 of 18 correct answers. Mean = average percent correct answers

^b^Average of the proportions and means for the three trials

^c^Average for control schools + adjusted difference based on individual participant meta-analysis. 95% CI account for uncertainty of the control odds as well as the odds ratios for proportions and the control mean as well as the mean difference for means. The values in this table differ slightly from values reported in the text, which are the observed proportions in the intervention schools

^d^3 cluster randomized trials and 244 schools were included for all six outcomes. The number of participants is the number that completed the CTH test after 1 year. The effective sample size, which accounts for clustering, is the original sample size divided by the “design effect” (Table S16)

^e^Grading of Recommendations Assessment, Development and Evaluation (GRADE) Working Group grades of evidence

⊕ ◯◯◯ Very low certainty: The research does not provide a reliable indication of the likely effect. The likelihood that the actual effect will be substantially different is very high

⊕  ⊕ ◯◯ Low certainty evidence: The research provides some indication of the likely effect. However, the likelihood that the actual effect will be substantially different is high

⊕  ⊕  ⊕ ◯ Moderate certainty: The research provides a good indication of the likely effect of a treatment. The likelihood that the actual effect of the treatment will not be substantially different is moderate

⊕  ⊕  ⊕  ⊕ High certainty: The research provides a very good indication of the likely effect of a treatment. The likelihood that the actual effect will be substantially different from this is low

^d^Downgraded due to missing outcome data, which resulted in wider confidence intervals and may have resulted in some degree of bias, although the sensitivity analyses suggest the point estimates are robust

^g^Downgraded due to missing outcome data and insufficient sample size

## Discussion

The effectiveness of the IHC secondary school intervention was sustained for at least 1 year, but the effects were smaller. Like the results just after the intervention, the odds ratios for students were heterogeneous across the three trials, with the largest relative effects in Rwanda. Students in the control schools in Rwanda also had the lowest scores, as was the case just after the intervention. The adjusted differences for the primary outcome (passing scores) varied from 31.8% in Rwanda to 20.8% in Kenya. Just after the intervention, the adjusted differences for passing scores were 37.2% in Rwanda and 27.3% in Kenya.

The certainty of the evidence after 1 year is less for the main outcomes for students because of missing outcome data; 27% of the students enrolled in the trials did not complete the CTH test at 1 year. Loss to follow-up was similar in control and intervention schools. Likely reasons for their not taking the test include changing schools and dropping out. Older students and girls were slightly more likely not to have taken the test in both control and intervention schools. It is uncertain whether there were important differences between control and intervention schools in the reasons why participants were lost to follow-up that would potentially have biased the results.

### Other evidence

The three trials included in this meta-analysis are the first large, randomized trials of a school-based intervention to teach adolescents to think critically about health interventions, and the only evaluation that has assessed long-term learning-retention [2, 3]. A systematic review of school-based interventions to enhance adolescents’ ability to critically appraise health claims found one randomized trial and seven non-randomized studies [3]. The authors concluded that “we know little about the effectiveness of educational interventions to teach adolescents critical appraisal skills.” A second systematic review of interventions to improve people’s understanding of key concepts for assessing the effects of health interventions found 14 randomized trials and 10 non-randomized studies [2]. Five of those studies were in schools (grades 7–12), and all those studies were included in the first review. An updated search using the same search strategies used in the second review up to March 2023 [28] found one additional non-randomized study of a school-based intervention (grades 7–12) [29].

### Process evaluations of the three trials

Process evaluations conducted alongside of the trials suggest that the teacher training workshop, completion of all the lessons, the design of the resources, the perceived value of the lessons, and administrative support contributed to the effectiveness of the intervention (Table 8) [30–32]. Inadequate time, the fact that the IHC lessons were not included in the national curricula and examinations, and the lack of printed materials for students may have impeded the effectiveness of the intervention.

**Table 8:** Synthesis of the main findings of process evaluations conducted alongside each of the three trials

Factors & potential impacts	Findings
**Facilitators**	
Teacher training workshop	Nearly all the teachers in intervention schools said the workshop was helpful or essential. They said it improved their understanding, motivation, and confidence to teach the lessons
Delivery of the lessons	Teachers reported completing all the lessons and using the educational resources with minimal adaptation. They sometimes used the local language instead of English and used some local examples instead of the ones provided in the lessons. Most students attended most of the lessons. The teachers said that the students achieved the lesson goals, apart from the lessons on random allocation and random error
Design of the resources	Teachers said the educational resources easy to access, understand, and use. They said most of the teaching strategies were familiar and easy to adapt, and a couple of teaching strategies were new to them and appreciated, such as use of response cards. Students said the lessons enjoyable and understandable
Value of the lessons	Teachers, students, and other stakeholders all said the lessons were relevant to daily life and valuable. Teachers and students said this motivated them. They said the lessons addressed skills that are important for students, teachers, and the public
Administrative support	Teachers in Kenya and Rwanda reported receiving support from their school’s administration, including time and resources to teach the lessons
**Barriers**	
Inadequate time	Teachers were unable to complete the lessons in a single 40-min period and generally used more time—sometimes as much as 120 min. Some teachers also reported not having enough time to prepare sufficiently due to competing demands on their time. The fact that it was an otherwise busy school term following school closures because of the COVID-19 pandemic may have contributed to this
Curricula and examinations	Students, teachers, curriculum developers, and education authorities all identified the lessons not being in the curriculum or national examinations as a major barrier to implementing and scaling up the intervention
Lack of printed material	Teachers, students, and some education authorities viewed the lack of a textbook or printed materials for students as a barrier to scaling up the intervention, since students lacked access to resources outside of the classroom

Process evaluations were conducted alongside each of the three trials included in this meta-analysis [30–32]. These findings were consistent across the three process evaluations

### Effect modifiers

Overall, 47% of students in the intervention schools did not have a passing score after 1 year compared to 42% just after the intervention. This indicates that they did not have a basic understanding of the nine key concepts included in the lessons and would need additional or alternative instruction [11]. Students in larger classes are less likely to have benefitted from the intervention, and students who performed poorly on end-of-term exams may be less likely to have benefitted. In addition, girls are probably less likely to have benefitted after 1 year.

It is uncertain why girls were less likely to have a passing score on the CTH test than boys. Gender inequality in educational outcomes differs between countries but persists in sub-Saharan Africa [33–35]. A possible explanation for girls performing less well than boys is that in countries where women have a lower social status, education may be perceived as less important for girls [33]. There also may have been biased instruction [34]. Another possible explanation is that for many girls in low-income countries, adolescence is a time of extreme vulnerability, when they are under pressure from social norms and cultural practices that place restrictions on them [36].

### Retention of what was learned

The effect of the IHC secondary school intervention on students’ and teachers’ ability to think critically about health choices persisted for at least 1 year. However, there was some decay in what was learned from the lessons. For students, there was about 12% decay in learning based on passing scores (adjusted for chance) and about 24% based on mastery scores. For teachers, there was about 4% and 13% decay based on passing and mastery scores respectively.

A large, randomized trial of the IHC primary school intervention also assessed outcomes after a year [37]. The primary school children in that trial retained what they learned for at least 1 year. Their scores were, in fact, better after 1 year. The difference in learning retention between the primary and secondary school interventions could reflect differences in the learners, the contexts, or the interventions. The primary school intervention included textbooks that used a comic book story to explain and illustrate the key concepts and exercise books for the children. It included nine 80-min lessons (about 12 h altogether). Each lesson included review of the previous lesson, reading the textbook out loud, discussion, an activity, and exercises [38]. The IHC secondary school intervention did not include resources that were handed out to the students. It included ten lessons designed to be taught in 40 min (about 7 h altogether), and 40 min frequently was not enough time to teach the lessons. Possible reasons why learning retention was better for the primary school intervention are that there may have been more active learning and engagement of the children [39] and more practice testing (using the exercise books) [40].

A review of retention of basic science knowledge suggests that decay in what was learned in school is common, with only two thirds to three fourths of knowledge being retained after 1 year [41]. To inform the choice of teaching strategies used in the IHC secondary school resources, we conducted an overview of systematic reviews of the effects of teaching strategies [42]. Only 21 of the 326 included reviews reported learning retention as an outcome. Among the 37 strategies that we considered most relevant to teaching students to think critically about health, only two were found to improve learning retention. Practice testing is probably more effective than restudying for retention of knowledge and skills [40], and signaling to attract the learners’ attention and highlight important information probably improves retention [43].

We used teaching strategies that could improve retention. In each lesson, we included questions about the previous lesson, which the teacher asked the class. We suggested using response cards, which probably increase participation [44]. We included a quiz in lessons 5 and 10, which were reviews of the previous lessons. The digital resources included printouts of the quizzes, but schools rarely printed materials for students due to the cost. We used signaling in the presentations included in the projector version of the lessons. We designed the lessons to include small group discussions, buzz groups (brief, intense discussions of a specific question with two or three people sitting next to each other), and class discussion to actively engage the students in learning. Teachers could decide which of these to use in each lesson. They often chose to use class discussion for several reasons. Small group discussion takes more time, both small group discussion and buzz groups can be difficult to monitor, and teachers were more familiar and comfortable with class discussions. The extent to which the teaching strategies used in the lessons affected retention of what was learned is uncertain since we do not have a reliable comparison.

### Use of what was learned

The value of our intervention, or any other educational intervention, is limited if learners are unable to transfer what they learn to other contexts. There is uncertainty about how to achieve and evaluate transfer of learning [45]. We assessed transfer or use of what was learned by students in the intervention schools based on self-report and observations by teachers in process evaluations and using a “diary,” as reported in this meta-analysis.

Students and teachers that participated in the process evaluations reported using at least some of the key concepts they learned in their daily lives [30–32]. Most of the IHC key concepts are applicable to many types of interventions unrelated to health, including environmental, educational, and social interventions [46]. In the process evaluations, students also reported applying what they learned to decisions unrelated to health.

Based on self-report, most students in the intervention schools used what they learned from the lessons a little (37%) or a lot (51%). Most teachers in the intervention schools also noticed their students using what they learned from the lessons sometimes (63%) or a lot (25%).

When asked to identify and assess a claim that they had encountered during the past week, students in the intervention schools were more likely to assess the reliability of that claim correctly (adjusted difference 16%), but only 23% of students in the intervention schools correctly identified and assessed the reliability of a claim about the effects of health actions (Table 5). We did not find substantial differences in the reasons that students gave for choosing whether to take or recommend the health action, including consideration of the reliability of the claim, advantages of the health action, advantages of the health action, personal experience, and health professional or researcher advice.

Other studies have shown that critical thinking can be learned in ways that promote transfer to contexts outside of the classroom [47]. However, available research evidence linking critical thinking to adult outcomes is limited and primarily based on associations [48]. Nonetheless, research has found consistent positive associations of modest size between cognitive competencies and desirable educational, career, and health outcomes. Higher levels of educational attainment are associated with reductions in adverse health events and increases in healthy behaviors. The association between education and health behaviors might be due in part to greater trust of science and general cognitive skills, including critical thinking, which enable people to make better-informed health decisions [48, 49].

### Adverse effects

Educational interventions can have undesirable as well as desirable effects, but potential adverse effects of educational interventions are rarely reported or even considered in evaluations of educational interventions [50, 51]. We assessed potential adverse effects of the IHC secondary school intervention by asking teachers to report any adverse events, qualitatively, and by asking students and teachers about potential adverse effects at 1 year. No adverse events were reported by teachers during the trial [7]. Based on preliminary findings from the process evaluations and interviews with teachers [51, 52], we identified adverse outcomes that we assessed quantitatively in this study, including wasted time, conflict due to students challenging the beliefs or choices of others, decision-making harms due to misunderstandings, and stress caused by the lessons.

Because we only assessed these outcomes in intervention schools, we are unable to estimate the extent to which they are attributable to the intervention. For example, although there is some evidence of inequities in the extent to which students benefited from the IHC secondary school intervention, we were unable to estimate the extent to which the intervention causes inequities. Similarly, we were unable to estimate the extent to which the intervention caused participants to waste time and resources. Based on self-report, 10% of students reported that what they learned from the *Be Smart about your Health* lessons was less helpful than what they learned in other subjects (Table S9b). This suggests that for those students, the intervention may have caused them to waste their time, but we cannot estimate the size of this potential adverse effect in comparison to the standard curriculum. Moreover, 64% of students reported that what they learned was more helpful than what they learned in other subjects.

The IHC secondary school intervention could cause conflict between students and others by causing students to question other people’s claims, beliefs, or choices, and people becoming irritated or defensive. Most (84%) of the teachers in intervention schools reported that students challenged things that they said because of the *Be Smart about your Health* lessons, but only 10% said they experienced this negatively. Nonetheless, this suggests that the intervention may cause conflict and that consideration should be given to mitigating this; for example, by introducing and practicing non-confrontational strategies for questioning the basis for claims.

Another potential adverse effect of the IHC secondary school intervention is poor choices due to misunderstanding. A misunderstanding that we explored was that some students appeared to believe that unreliable claims that were used as examples in the lessons were reliable. Half (50%) of the teachers in intervention schools reported observing their students relying on unreliable claims that were used as examples in the lessons. We cannot quantify how many students did this or how often. Nonetheless, consideration should be given to mitigating misunderstandings of unreliable claims; for example, by ensuring clear communication about the reliability of the claims that are used, reiterating when a claim is unreliable, and reiterating that the focus of the lessons is on the key concepts, not on the examples that are used.

Some (18%) of the students reported that the *Be Smart about your Health* lessons were stressful or very stressful, but it is uncertain how this compares to other subjects or what caused the lessons to be stressful. For example, the lessons were an add-on to the standard curriculum and took time away from other subjects and preparing for examinations in those subjects. The extent to which this caused the lessons to be stressful is uncertain. Some teachers said preparing and teaching the lessons stressful. The most common reasons that they gave for this were that this took time away from other subjects and completing the lessons during the time available was stressful.

### Limitations

A limitation of this meta-analysis is that the same research team was responsible for the meta-analysis, the included trials, the design of the intervention, and development of the outcome measure. To avoid bias in the assessments of the risk of bias and the certainty of the evidence, two researchers who were not involved in the trials made these assessments when the results just after the intervention were published [12]. The same independent researchers assessed the risk of bias and certainty of the evidence for the 1-year follow-up results. There was a moderate risk of bias for all the main outcomes because of loss to follow-up (Table S15) and lower certainty of the evidence (Table S16).

The questions we used to assess transfer and the rubric used to code their responses were not validated. We pilot tested the questions, and the overall results are congruent with the results of the CTH test. However, due to the burden of coding the answers to those questions, we only asked 10 students from each school to answer those questions. Consequently, there is insufficient data to reliably estimate the effects for each trial (country). In addition, although the data were coded independently by two people in each country, there may be differences between countries in how the coding was implemented.

This was the first time that teachers in the three trials taught the lessons, and the lessons were an add-on to the standard curriculum. Also, the lessons were taught in the first or second term when schools re-opened following closures due to the COVID-19 pandemic. This might have created additional stress for teachers and students. If the teachers had more experience, the lessons were in the curriculum, and there were normal circumstances, they might be more effective.

Nonetheless, the results after 1 year suggest that additional lessons are likely needed to reinforce what was learned, improve long-term learning-retention, and improve use of what was learned. It also is likely that more than seven hours of classroom time are needed as well as additional lessons to ensure that all or nearly all the students benefit. Moreover, these lessons focused on just nine key concepts. Additional lessons are needed to teach other key concepts [6].

### Implications

The results of this research show that adolescents in secondary schools in low-income countries with limited resources can learn valuable skills needed to decide what to believe or do in relation to health claims. This could help reduce susceptibility to misinformation about health interventions [53, 54]. It also could help students to make better decisions about healthcare as they grow older, thereby reducing waste and unnecessary suffering [55]. However, there are multiple other factors that limit the potential impacts on healthcare decisions and health, including a lack of access to health services and to reliable information [56]. It is not easy to find evidence-based information, and there is a tremendous amount of misinformation [54, 57, 58]. In low-income countries where many people have limited if any access to the Internet and to health professionals, accessing reliable information is especially challenging.

Inequities in the extent to which students benefit from the intervention might be reduced by translating the lessons for students lacking English reading proficiencies. Other strategies that might help include providing students with printed resources, more use of formative assessments and feedback, smaller classes, and teacher training focused on strategies for supporting students who need additional help [59]. However, inequities in the outcomes of the IHC secondary school intervention likely reflect broader inequities in educational outcomes shaped by educational and other societal systems [33], and interventions to reduce educational inequities may need to start early in children’s lives [60].

Scaling up the intervention is likely to depend on incorporation of the IHC key concepts into the curriculum and examinations. The resources are freely available, are easily accessible using a web browser, and can be used offline. However, use of the resources in contexts outside of East Africa should be tested and may require translation and adaptation [61].

Additional lessons are needed to reinforce what was learned and to teach the ability to apply other key concepts. Human-centered design can help to ensure that resources for those lessons are experienced positively [4] and that they are effective. Ideally, children should start learning to think critically about health interventions and other interventions [46] as young as possible, and lessons should progress throughout their education, reinforcing concepts, skills, and dispositions that were learned and introducing new ones [62].

## Supplementary Information

Additional file: Box S1. Key concepts included in the IHC lower-secondary school resources. Box S2. Descriptions of the contexts in which the trials were conducted. Table S1. Eligibility criteria. Table S2. Inclusion and exclusion criteria for the included trials. Table S3. Secondary outcomes. Table S4. Potential effect modifiers. Table S5. Sensitivity analyses. Table S6. Intended behaviors. Table S7. Self-efficacy. Table S8. Intervention school students’ views of the lessons. Tables S9a to S9c. Intervention school students – transfer and adverse effects. Tables S10a to S10i. Transfer of what was learned to daily life. Tables S11a to S11g. Intervention school teachers – transfer and adverse effects. Table S12a to S12d. Potential effect modifiers. Table S13. Credibility of the effect modifier analyses for the primary outcome. Table S14a to 14c. English reading proficiency subgroup analyses. Table S15. Risk of bias assessment. Table S16. GRADE evidence profile. Fig. S1. Students with a mastery score (≥14 out of 18) at 1-year follow-up. Fig. S2. Students’ mean score at 1-year follow-up. Fig. S3. Teachers with a passing score. Fig. S4. Teachers with a mastery score. Fig. S5. Teachers’ mean score. Appendix 1. GREET checklist. Appendix 2. The Critical Thinking about Health test. Appendix 3. Additional questions about transfer and adverse effects. Appendix 4. Critical thinking about health diary. Appendix 5. Rubric for scoring the diary.

## Data Availability

Data collected for this meta-analysis, including deidentified individual participant data and a data dictionary defining each field in the set, will be made freely available with publication on Zenodo, licensed under a Creative Commons Attribution 4.0 International license. The study protocol was published on Zenodo prior to recruiting participants: https://zenodo.org/record/6597493#.ZC-2xXZBzq4. The *Be Smart about your Health* resources are licensed under a Creative Commons Attribution-Non-Commercial-Share-Alike 4.0 International license and are freely available: https://besmarthealth.org/.
