# Supplementary material for: Effects of the informed health choices secondary school intervention after 1 year: a prospective meta-analysis using individual participant data
Source: Trials. 2024 Oct 30;25:733. doi: 10.1186/s13063-024-08577-w (PMC11523815; doi:10.1186/s13063-024-08577-w)
Supplement: Supplementary file 1 — Additional file: Box S1. Key concepts included in the IHC lower-secondary school resources. Box S2. Descriptions of the contexts in which the trials were conducted. Table S1. Eligibility criteria. Table S2. Inclusion and exclusion criteria for the included trials. Table S3. Secondary outcomes. Table S4. Potential effect modifiers. Table S5. Sensitivity analyses. Table S6. Intended behaviors. Table S7. Self-efficacy. Table S8. Intervention school students’ views of the lessons. Tables S9a to S9c. Intervention school students – transfer and adverse effects. Tables S10a to S10i. Transfer of what was learned to daily life. Tables S11a to S11g. Intervention school teachers – transfer and adverse effects. Table S12a to S12d. Potential effect modifiers. Table S13. Credibility of the effect modifier analyses for the primary outcome. Table S14a to 14c. English reading proficiency subgroup analyses. Table S15. Risk of bias assessment. Table S16. GRADE evidence profile. Fig. S1. Students with a mastery score (≥14 out of 18) at 1-year follow-up. Fig. S2. Students’ mean score at 1-year follow-up. Fig. S3. Teachers with a passing score. Fig. S4. Teachers with a mastery score. Fig. S5. Teachers’ mean score. Appendix 1. GREET checklist. Appendix 2. The Critical Thinking about Health test. Appendix 3. Additional questions about transfer and adverse effects. Appendix 4. Critical thinking about health diary. Appendix 5. Rubric for scoring the diary. [file 13063_2024_8577_MOESM1_ESM.docx]

Supporting information

**Effects of the Informed Health Choices secondary school intervention: a prospective meta-analysis of one-year follow-up data**

Box S1. Key concepts included in the IHC lower-secondary school resources 2

Box S2. Descriptions of the contexts in which the trials were conducted 3

Table S1. Eligibility criteria 4

Table S2. Inclusion and exclusion criteria for the included trials 5

Table S3. Secondary outcomes 6

Table S4. Potential effect modifiers 7

Table S5. Sensitivity analyses 8

Table S6. Intended behaviors 9

Table S7. Self-efficacy 10

Table S8. Intervention school students' views of the lessons 11

Tables S9a to S9c. Intervention school students – transfer and adverse effects 12

Tables S10a to S10i. Transfer of what was learned to daily life 15

Tables S11a to S11g. Intervention school teachers – transfer and adverse effects 18

Table S12a to S12d. Potential effect modifiers 21

Table S13. Credibility of the effect modifier analyses for the primary outcome 25

Table S14a to 14c. English reading proficiency subgroup analyses 26

Table S15. Risk of bias assessment 29

Table S16. GRADE evidence profile 30

Figure S1. Students with a mastery score (≥14 out of 18) at one-year follow-up 32

Figure S2. Students’ mean score at one-year follow-up 33

Figure S3. Teachers with a passing score 34

Figure S4. Teachers with a mastery score 35

Figure S5. Teachers' mean score 36

Appendix 1. GREET checklist 37

Appendix 2. The Critical Thinking about Health test 41

Appendix 3. Additional questions about transfer and adverse effects 68

Appendix 4. Critical thinking about health diary 75

Appendix 5. Rubric for scoring the diary 79

# Box S1. Key concepts included in the IHC lower-secondary school resources

| The *Informed Health Choices Key Concepts* are principles for thinking critically about whether to believe claims about health actions and for deciding what to do.* These principles were the starting point for developing these resources. These nine concepts, which are the focus of *Be Smart about your Health* resources were chosen from a list of 49 concepts that has been developed over many years by researchers and others around the world.   1. Health actions can have helpful effects, but they can also have harmful effects and be expensive. 2. The effects of most health actions are not obvious, especially changes that do not occur right after the health action. 3. Usually, personal experience (something that happened to someone after taking a health action) is a weak basis for claims about the effects of health actions. 4. Health actions that have not been evaluated in a reliable comparison but are commonly-used or have been used for a long time are often assumed to work. However, they might not work and might be harmful or wasteful. 5. Health actions that have not been evaluated in a reliable comparison but are new, expensive, or technologically impressive are often assumed to work. However, they also might not work and might be harmful or wasteful. 6. Knowledge about the effects of health actions depends on comparisons. 7. In a comparison between health actions, important differences (other than the health actions) between comparison groups can be misleading. Randomly creating groups makes sure groups of people are as similar as possible at the start of a comparison and avoids unknown differences. 8. If a comparison between health actions is too small, we cannot be sure that the results reflect a true difference (or lack of difference) between the effects of the different health actions. The results could just be by chance. 9. People making a choice about whether to take a health action should consider the potential benefits and potential harms, costs, and other advantages and disadvantages. People making a community choice should also consider who will benefit, who will be harmed, who will achieve savings, and who will bear the costs. |
| --- |

* More information about the Informed Health Choices Key Concepts can be found here: <https://www.informedhealthchoices.org/key-concepts/>.

# Box S2. Descriptions of the contexts in which the trials were conducted

| **Kenya**  The Kenyan trial included a representative sample of public and private secondary schools in Kisumu County that followed the national curriculum. There are four years of secondary school education in Kenya (following 8 years of primary school), with three school terms each year. Each term is normally 10-13 weeks. Students typically enroll when they are about 14 years old and graduate when they are about 18. Class size in public secondary schools is generally 40–59 students per class.  About 63% of Kisumu secondary schools had at least one laptop or desktop computer, 35% of the schools owned a projector, and 17% had Internet access. Students had limited access to computers and few students owned phones.  Teachers in Kenya qualify after undergoing training by an accredited university or training institute. Nearly 95% of secondary school teachers hold a bachelor’s degree and about 2% have a post-graduate degree.  The curriculum was knowledge-based. Teaching was exam-oriented and critical thinking was not assessed in national examinations. Health was a topic in nine different subjects. The government plans to implement a new, competency-based curriculum by 2024. “Critical thinking and problem solving” are one of seven core competencies in the framework for the new curriculum. The core competencies cut across subjects. In the proposed curriculum, health education is a subject.  **Rwanda**  The Rwandan trial included a representative sample of public, private, and government-aided secondary schools in two districts from each of the five provinces of Rwanda (10 districts total). There are six years of secondary school in Rwanda (following six years of primary school). The first three years are lower-secondary. There are three school terms each year. Each term is 12-14 weeks. The official age range is 13-18 for secondary school and 13-15 for lower-secondary. The average number of students per classroom in secondary schools is 39.  Most secondary schools had computers (86%) and grid electricity supply (77%). Two thirds had “smart classrooms”, which included student computers, a projector, smart boards, and Internet access.  Lower-secondary teachers must have at least a Diploma in Education - a credential earned after three years of post-secondary study that prepares them to teach two subjects. Upper-secondary school teachers must have a four-year bachelor’s degree in education.  A competence-based curriculum was implemented in Rwanda in 2016. All subjects include generic competences for higher order thinking. The generic competences in the curriculum are critical thinking, creativity and innovation, research and problem solving, communication, co-operation, interpersonal relations, life skills, and lifelong learning. Health is a topic in three subjects: biology and health sciences, home science, and English (in which health topics are used as a context for teaching English).  **Uganda**  The Ugandan trial included a representative sample of public and private secondary schools that followed the national curriculum in six districts of Uganda (Luweero, Wakiso, Mpigi, Mukono, Kampala, and Kayunga). Secondary education in Uganda is six years (following seven years of primary school). Lower-secondary (“Ordinary Level”) is four years. There are three school terms each year that normally last about 12 weeks. The official entry age of lower-secondary education is 13 years. The average class size is 69.  Most schools had at least one computer (87%) and hydroelectric power (71%), and 40% had a projector. Most schools did not have Internet access.  Teacher training at National Teachers Colleges requires two years of study. Successful trainees are awarded Diplomas in Secondary Education. Universities also offer undergraduate teaching programs of three to four years in length. The government is currently phasing out teaching diplomas in favor of a bachelor’s degree in education.  Uganda’s National Curriculum Development Centre introduced a new competence-based curriculum for lower-secondary school students in 2020. Critical thinking is one of seven generic skills in the new curriculum. Several others, such as problem solving, are related to critical thinking. The generic skills are taught across subjects. Health is a topic in biology, physical education, and nutrition and food technology. |
| --- |

# Table S1. Eligibility criteria

|  | **Criteria*** |
| --- | --- |
| **Participants** | Students in secondary schools and their teachers |
| **Intervention** | The IHC secondary school intervention (providing a teacher training workshop and the Be Smart about your Health resources) as an add-on to the standard national curriculum |
| **Comparison** | The standard national curriculum in the country where the trial is conducted, without intervening |
| **Outcomes** | Outcomes measured using the Critical Thinking about Health test, including the proportion of students with a passing score (the primary outcome), the average score on the test, the proportion of students with a score that indicates mastery of the nine concepts, self-efficacy, intended behaviors, and teachers’ ability to understand and apply the nine concepts. |
| **Study design** | Cluster-randomized trials |

* The eligibility criteria for this meta-analysis are shared by all three of the trials.

# Table S2. Inclusion and exclusion criteria for the included trials

|  |  | **Kenya** | **Rwanda** | **Uganda** |
| --- | --- | --- | --- | --- |
| **Schools** | **Inclusion criteria** | Public or private schools that follow the national curriculum | Public, private, or government aided schools that follow the national curriculum | Public or private schools that follow the national curriculum |
|  | **Exclusion criteria** | Schools that participated in the user testing or piloting of the resources, special needs schools, international schools, and schools in districts prone to floods and insecurity | Schools without an Internet connection, schools with less than 100 students or 10 teachers, schools that participated in the user testing and piloting of the intervention, and special needs schools, and schools that are hard to reach | Schools that participated in the user testing and piloting of the resources, special needs schools, international schools, and adult-only schools |
|  | **Stratification variables for random selection of eligible schools and random allocation of included schools** | Sub-counties (districts) in Kisumu County, ownership (private vs public), and geographical location (rural vs urban) | Districts in all five provinces and performance (low vs high as defined by the National Examination and School Inspection Authority) | Districts in the central region, ICT access (blackboard only vs blackboard and a projector), and school ownership (private vs public). |
| **Teachers** | **Inclusion criteria** | Form (level) one teachers selected by the principal, with a smartphone or laptop computer, who teach a subject related to health or critical thinking | Level two (senior 2) teachers that teach biology and health sciences, physics, chemistry, or mathematics | Level two (lower secondary level) teachers that teach biology or health sciences |
|  | **Exclusion criteria** | None | None | None |
| **Students** | **Inclusion criteria** | Form (level) one (year 7) students (normal starting age 14) | Level two (year 8) students (normal starting age 13) | Level two (year 8) students (normal starting age 14) |
|  | **Exclusion criteria** | Students that chose not to participate | Students that chose not to participate | None |

# Table S3. Secondary outcomes

| Outcome | Measurement* | Participants† | Variable type |
| --- | --- | --- | --- |
| Passing score (a basic understanding of the nine key concepts and how to apply them) | ≥ 9 correct answers out of 18 MCQs | teachers‡ | dichotomous |
| Mastery score (mastery of the nine key concepts | ≥ 14 correct answers out of 18 MCQs | students and teachers | dichotomous |
| Mean score | percent correct answers out of 18 MCQs | students and teachers | continuous |
| Mastery of each of the nine key concepts | both MCQs for the concept answered correctly | students | dichotomous |
| Intended behaviors | 3 questions about how likely the participant would be to do something | students | categorical (dichotomized as the proportion of ‘easy’ or ‘very easy’ responses in the analysis) |
| Self-efficacy (confidence in one’s own ability) | questions about how difficult or easy 4 actions are for the participant | students | categorical (dichotomized as the proportion of ‘likely’ or ‘very likely’ in the analysis) |
| Views of the lessons | 3 questions about how much the participant liked the lessons, how easy they were to understand, and how helpful they were | students§ | categorical (also reported dichotomously as, e.g., ‘helpful’ or ‘very helpful’) |

MCQs = multiple-choice questions

* All the secondary outcomes were measured using the Critical Thinking about Health (CTH) test. Each MCQ had 3 response options. The questions about intended behaviors, self-efficacy, and views of the lessons had Likert response options.

† Students and teachers in both intervention and control schools completed the CTH test

‡ Passing scores for students was the primary outcome

§ These questions were only included in the CTH test for students in the intervention schools

# Table S4. Potential effect modifiers

| **Hypothesis** | **Rationale** |
| --- | --- |
| The effects will be larger in schools using the projector version of the resources than in schools using the blackboard version. | The projector version of the resources provides scaffolding (support) for teachers who will be teaching critical thinking about health choices for the first time. The presentations help to ensure that the key messages are clearly communicated and understood by the students, and they include text and illustrations that are not available to students in schools using the blackboard version. |
| The effects will be larger in schools with a smaller class size | It is easier for teachers to monitor, assess, and engage all the students when there are fewer students in a classroom. In classrooms with a large number of students, some students may not engage, there is less time for individual students to participate in classroom discussions, and it is more difficult for teachers to manage small group discussions. |
| The effects will be larger for students who perform better on examinations. | We designed the lessons to benefit all students. Nonetheless, students who perform well on examinations are likely to benefit more than other students. Students who perform well on examinations may be more likely to have higher English proficiency, better study habits, better home environments, better cognitive and meta-cognitive skills, and better test-taking skills. |

# Table S5. Sensitivity analyses

|  | **Control Schools** | **Intervention Schools** | **Adjusted difference** | **Odds ratio** | **p** | **ICC** | |
| --- | --- | --- | --- | --- | --- | --- | --- |
|  | 3942 students*  122 schools | 4356 students*  122 schools |  |  |  | **Trial** | **School** |
| **Primary outcome** |  |  |  |  |  |  |  |
| Students with a passing score (≥9/18) | 1123／3918 (28.7%) | 2273／4324 (52.6%) | 25.3% (15.7–35.0) | 3.7 (1.8–7.7) | 0.0004 | 0.005 | 0.175 |
| **Secondary outcomes** |  |  |  |  |  |  |  |
| **Students** |  |  |  |  |  |  |  |
| Students with a mastery score (≥14/18) | 93／3918 (2.4%) | 672／4324 (15.5%) | 12.0% (10.0–14.1) | 8.4 (5.9–11.8) | <0.0001 | <0.001 | 0.262 |
| Mean score for students | 38.8% (16.8) | 50.7% (21.5) | 12.1% (8.0–16.3) |  | <0.0001 | <0.001 | 0.172 |
| Lee bounds |  |  | 11.1% to 12.5%  (4.4% to 18.8%) |  |  |  |  |
| **Teachers**^†^ |  |  |  |  |  |  |  |
| Teachers with a passing score (≥9/18) | 64／104 (61.5%) | 101／108 (93.5%) | 31.8% (15.3–48.3) | 10.9 (4.4–27.2) | <0.0001 | 0.165 |  |
| Teachers with a mastery score (≥14/18) | 13／104 (12.5%) | 79／108 (73.1%) | 60.9% (47.5–74.2) | 24.7 (11.0–55.2) | <0.0001 | 0.116 |  |
| Mean score for teachers | 54.0% (18.9) | 80.7% (15.8) | 27.0% (22.6–31.3) |  | <0.0001 | 0.113 |  |
| Lee bounds |  |  | 26.0% to 28.5%  (21.1% to 34.2%) |  |  |  |  |

Individual participant data meta-analyses with inverse probability weighting for student outcomes and Lee bounds for mean scores for students and teachers.

Data are n／N (%), % (95% CI), or % (SD). Clustering was accounted for using random intercepts at the level of trial and, for outcomes measured on students, random intercepts at the level of school within trial. Logistic regression was used to estimate adjusted odds ratios for passing and mastery, which are re-expressed as adjusted differences. Linear regression was used to estimate adjusted differences in mean scores. Outcomes for students lost to follow-up were assumed to be missing at random (MAR), which was accounted for using inverse probability weighting. Outcomes for teachers lost to follow-up were assumed to be missing completely at random (MCAR). Intraclass correlation coefficients (ICCs) are estimated at the same levels as the random intercepts (trial and school within trial). Fixed effects were used to adjust all estimates as described in the Methods section. Wald-type confidence intervals and two-sided p-values were computed in all analyses.

* 24 students in control schools and 32 students in intervention schools were not included in the analyses because of incomplete performance data. The analyses are adjusted for performance, so complete data on this variable is required.

^†^ Since there was only one teacher per school, inverse probability weighting is not possible for teachers and the results shown here are identical to the results shown in Table 2.

# Table S6. Intended behaviors

***Think about an illness that you might get. Imagine someone claiming (saying) that a particular treatment might help you get better.***

|  | **How likely are you to find out what the claim was based on (for example by asking the person making the claim)?** | | **How likely are you to find out if the claim was based on a research study comparing the treatment to no treatment?** | | | **How likely are you to say “yes” if you are asked to participate in a research study comparing two treatments for your sickness?** | | | |
| --- | --- | --- | --- | --- | --- | --- | --- | --- | --- |
|  | Control schools | Intervention schools | | Control schools | Intervention schools | | Control schools | Intervention schools |  |
|  | (3942 students) | (4356 students) | | (3942 students) | (4356 students) | | (3942 students) | (4356 students) |  |
| **Missing** | 2 (0.1%) | 1 (0.0%) | | 0 (0.0%) | 2 (0.0%) | | 1 (0.0%) | 8 (0.2%) |  |
| **I don't know** | 260 (6.6%) | 190 (4.4%) | | 384 (9.7%) | 230 (5.3%) | | 332 (8.4%) | 194 (4.5%) |  |
| **Very unlikely** | 472 (12.0%) | 450 (10.3%) | | 709 (18.0%) | 617 (14.2%) | | 597 (15.1%) | 697 (16.0%) |  |
| **Unlikely** | 957 (24.3%) | 950 (21.8%) | | 1000 (25.4%) | 1025 (23.5%) | | 633 (16.1%) | 763 (17.5%) |  |
| **Likely** | 1363 (34.6%) | 1630 (37.4%) | | 1054 (26.7%) | 1398 (32.1%) | | 1012 (25.7%) | 1238 (28.4%) |  |
| **Very likely** | 888 (22.5%) | 1135 (26.1%) | | 795 (20.2%) | 1084 (24.9%) | | 1366 (34.7%) | 1456 (33.4%) |  |
| **Likely or very likely** | 2251 (57.1%) | 2765 (63.5%) | | 1849 (46.9%) | 2482 (57.0%) | | 2378 (60.3%) | 2694 (61.8%) |  |
| **Adjusted odds ratio*** |  |  | |  |  | |  |  |  |
| **One year follow-up** | 1.3 (95% CI 1.0 to 1.6) | | 1.5 (95% CI 1.2 to 1.9) | | | 1.1 (95% CI 1.0 to 1.1) | | | |
| **Initial^†^** | 1.1 (95% CI 1.0 to 1.2) | | 1.5 (95% CI 1.4 to 1.7) | | | 1.0 (95% CI 0.9 to 1.1) | | | |
| **Adjusted difference^‡^** |  | |  | | |  | | | |
| **One year follow-up** | 5.7% (95% CI 0.2% to 11.2%) | | 10.1% (95% CI 5.2% to 15.0%) | | | 1.9% (95% CI 0.8% to 3.0%) | | | |
| **Initial^†^** | 2.3% (95% CI -0.0% to 4.6%) | | 10.7% (95% CI 8.3% to 13.0%) | | | 0.3% (95% CI -2.3% to 3.0%) | | | |

* Mixed-effects logistic regression was used to estimate adjusted odds ratios. Estimates are adjusted for school ownership (public or government-funded versus private), use of a projector (versus blackboard), and school performance based on examinations taken prior to the intervention (low versus moderate or high). Hierarchical random intercepts were used to account for clustering of student within school and school within trial (country).

† Just after the intervention.

^‡^ Odds ratios are re-expressed as adjusted differences.

# Table S7. Self-efficacy

***How difficult or easy would you find each of these actions to be?***

|  | **How difficult or easy do you find knowing if a claim about a treatment is based on a research study comparing treatments?** | | **How difficult or easy do you think it is to find information about treatments that is based on research studies comparing treatments?** | | **How difficult or easy do you find judging the trustworthiness of the results of a research study comparing treatments?** | | **How difficult or easy do you find knowing if the results of a research study comparing treatments are relevant to you?** | |
| --- | --- | --- | --- | --- | --- | --- | --- | --- |
|  | **Control**  **schools** | **Intervention**  **schools** | **Control**  **schools** | **Intervention**  **schools** | **Control**  **schools** | **Intervention**  **schools** | **Control**  **schools** | **Intervention**  **schools** |
| **Missing** | 2 (0.1%) | 6 (0.1%) | 3 (0.1%) | 1 (0.0%) | 19 (0.5%) | 6 (0.1%) | 3 (0.1%) | 5 (0.1%) |
| **I don't know** | 369 (9.4%) | 287 (6.6%) | 292 (7.4%) | 246 (5.6%) | 400 (10.1%) | 295 (6.8%) | 335 (8.5%) | 288 (6.6%) |
| **Very difficult** | 975 (24.7%) | 776 (17.8%) | 815 (20.7%) | 721 (16.6%) | 962 (24.4%) | 869 (19.9%) | 684 (17.4%) | 528 (12.1%) |
| **Difficult** | 1280 (32.5%) | 1202 (27.6%) | 1149 (29.1%) | 1342 (30.8%) | 1227 (31.1%) | 1414 (32.5%) | 941 (23.9%) | 1004 (23.0%) |
| **Easy** | 876 (22.2%) | 1413 (32.4%) | 1159 (29.4%) | 1302 (29.9%) | 870 (22.1%) | 1189 (27.3%) | 1229 (31.2%) | 1586 (36.4%) |
| **Very easy** | 440 (11.2%) | 672 (15.4%) | 524 (13.3%) | 744 (17.1%) | 463 (11.7%) | 583 (13.4%) | 750 (19.0%) | 945 (21.7%) |
| **Easy or very easy** | 1316 (33.4%) | 2085 (47.9%) | 1683 (42.7%) | 2046 (47.0%) | 1333 (33.8%) | 1772 (40.7%) | 1979 (50.2%) | 2531 (58.1%) |
| **Adjusted odds ratio*** | | | | | | | | |
| **One year** | 1.8 (95% CI 1.3 to 2.3) | | 1.2 (95% CI 1.0 to 1.4) | | 1.4 (95% CI 1.1 to 1.7) | | 1.4 (95% CI 1.2 to 1.6) | |
| **Initial^†^** | 1.7 (95% CI 1.5 to 1.9) | | 1.2 (95% CI 1.1 to 1.3) | | 1.8 (95% CI 1.5 to 2.1) | | 1.2 (95% CI 1.1 to 1.3) | |
| **Adjusted difference^‡^** | | | | | | | | |
| **One year** | 13.0% (95% CI 6.5% to 19.5%) | | 4.1% (95% CI -0.7% to 8.9%) | | 6.9% (95% CI 2.0% to 11.8%) | | 8.0% (95% CI 4.9% to 11.0%) | |
| **Initial^†^** | 12.4% (95% CI 9.4% to 15.3%) | | 4.1% (95% CI 1.5% to 6.8%) | | 12.0% (95% CI 8.7% to 15.2%) | | 4.9% (95% CI 2.5% to 7.3%) | |

Control schools N = 3942, Intervention schools N = 4356

* Mixed-effects logistic regression was used to estimate adjusted odds ratios. Estimates are adjusted for school ownership (public or government-funded versus private), use of a projector (versus blackboard), and school performance based on examinations taken prior to the intervention (low versus moderate or high). Hierarchical random intercepts were used to account for clustering of student within school and school within trial (country).

^†^ Just after the intervention.

^‡^ Odds ratios are re-expressed as adjusted differences.

# Table S8. Intervention school students' views of the lessons

| **How much did you like or dislike the lessons?** | | **How easy or difficult were these lessons to understand?** | | **How helpful or unhelpful has what you have learned been to you?** | |
| --- | --- | --- | --- | --- | --- |
| **Liked very much** | 3043 (69.9%) | **Very difficult** | 411 (9.4%) | **Very helpful** | 1740 (39.9%) |
| **Liked a little** | 874 (20.1%) | **Difficult** | 894 (20.5%) | **Helpful** | 1493 (34.3%) |
| **Disliked a little** | 223 (5.1%) | **Easy** | 2358 (54.1%) | **Unhelpful** | 1001 (23.0%) |
| **Disliked a lot** | 184 (4.2%) | **Very easy** | 671 (15.4%) | **Very helpful** | 100 (2.3%) |
| **Like a little or very much** | | **Easy or very easy** | | **Helpful or very helpful** | |
| **One year** | 3917 / 4356 (89.9%) |  | 3029 / 4356 (69.5%) |  | 3233 / 4356 (74.2%) |
| **Initial*** | 5425 / 5913 (91.7%) |  | 4281 / 5913 (72.4%) |  | 5506 / 5913 (93.1%) |

* Just after the intervention.

# Tables S9a to S9c. Intervention school students – transfer and adverse effects

**Table S9a. Helpfulness of what was learned - students' self-report**

Think about what you learned from these lessons. How helpful or unhelpful has that been to you?

|  | Kenya | | Rwanda | | Uganda | | Total | |
| --- | --- | --- | --- | --- | --- | --- | --- | --- |
|  | n | % | n | % | n | % | n | % |
|  | 1367 |  | 1251 |  | 1749 |  | 4367 |  |
| Missing | 5 | 0.4% | 24 | 1.9% | 0 | 0.0% | 29 | 0.7% |
| Very helpful | 822 | 60.1% | 727 | 58.1% | 1,151 | 65.8% | 2700 | 61.8% |
| Helpful | 439 | 32.1% | 330 | 26.4% | 498 | 28.5% | 1267 | 29.0% |
| Unhelpful | 63 | 4.6% | 105 | 8.4% | 49 | 2.8% | 217 | 5.0% |
| Very unhelpful | 38 | 2.8% | 65 | 5.2% | 51 | 2.9% | 154 | 3.5% |
| Helpful or very helpful | 1261 | 92.2% | 1057 | 84.5% | 1649 | 94.3% | 3967 | 90.8% |

**Table S9b. Helpfulness of what was learned compared to other subjects – students’ self-report**

Compared to what you learned in other subjects, how helpful has what you learned in these lessons been to you?

|  | Kenya | | Rwanda | | Uganda | | Total | |
| --- | --- | --- | --- | --- | --- | --- | --- | --- |
|  | n | % | n | % | n | % | n | % |
|  | 1367 |  | 1251 |  | 1749 |  | 4367 |  |
| Missing | 2 | 0.1% | 29 | 2.3% | 0 | 0.0% | 31 | 0.7% |
| Less helpful | 103 | 7.5% | 185 | 14.8% | 134 | 7.7% | 422 | 9.7% |
| About the same | 356 | 26.0% | 323 | 25.8% | 442 | 25.3% | 1121 | 25.7% |
| More helpful | 906 | 66.3% | 714 | 57.1% | 1173 | 67.1% | 2793 | 64.0% |

**Table S9c. Use of what was learned by students – students’ self-report**

How much have you used what you learned from the lessons?

|  | Kenya | | Rwanda | | Uganda | | Total | |
| --- | --- | --- | --- | --- | --- | --- | --- | --- |
|  | n | % | n | % | n | % | n | % |
|  | 1367 |  | 1251 |  | 1749 |  | 4367 |  |
| Missing | 7 | 0.5% | 29 | 2.3% | 0 | 0.0% | 36 | 0.8% |
| Not at all | 152 | 11.1% | 230 | 18.4% | 114 | 6.5% | 496 | 11.4% |
| A little | 360 | 26.3% | 467 | 37.3% | 797 | 45.6% | 1624 | 37.2% |
| A lot | 848 | 62.0% | 525 | 42.0% | 838 | 47.9% | 2211 | 50.6% |
| A little or a lot | 1208 | 88.4% | 992 | 79.3% | 1635 | 93.5% | 3835 | 87.8% |

**Table S9d. Conflict with teachers – students’ self-report**

If you challenged YOUR TEACHERS, how did this make you feel?

|  | Kenya | | Rwanda | | Uganda | | Total | |
| --- | --- | --- | --- | --- | --- | --- | --- | --- |
|  | n | % | n | % | n | % | n | % |
|  | 1367 |  | 1251 |  | 1749 |  | 4367 |  |
| Missing | 5 | 0.4% | 25 | 2.0% | 9 | 0.5% | 39 | 0.9% |
| I did not challenge | 155 | 11.3% | 387 | 30.9% | 411 | 23.5% | 953 | 21.8% |
| Very bad | 160 | 11.7% | 130 | 10.4% | 58 | 3.3% | 348 | 8.0% |
| Bad | 117 | 8.6% | 76 | 6.1% | 70 | 4.0% | 263 | 6.0% |
| Neither bad nor good | 260 | 19.0% | 177 | 14.1% | 305 | 17.4% | 742 | 17.0% |
| Good | 350 | 25.6% | 233 | 18.6% | 528 | 30.2% | 1111 | 25.4% |
| Very good | 320 | 23.4% | 223 | 17.8% | 368 | 21.0% | 911 | 20.9% |
| Bad or very bad | 277 | 20.3% | 206 | 16.5% | 128 | 7.3% | 611 | 14.0% |

**Table S9e. Conflict with parents or other adults – students’ self-report**

If you challenged YOUR PARENTS OR OTHER ADULTS AT HOME, how did this make you feel?

|  | Kenya | | Rwanda | | Uganda | | Total | |
| --- | --- | --- | --- | --- | --- | --- | --- | --- |
|  | n | % | n | % | n | % | n | % |
|  | 1367 |  | 1251 |  | 1749 |  | 4367 |  |
| Missing | 8 | 0.6% | 30 | 2.4% | 7 | 0.4% | 45 | 1.0% |
| I did not challenge | 167 | 12.2% | 274 | 21.9% | 299 | 17.1% | 740 | 16.9% |
| Very bad | 229 | 16.8% | 112 | 9.0% | 93 | 5.3% | 434 | 9.9% |
| Bad | 159 | 11.6% | 120 | 9.6% | 151 | 8.6% | 430 | 9.8% |
| Neither bad nor good | 289 | 21.1% | 221 | 17.7% | 297 | 17.0% | 807 | 18.5% |
| Good | 255 | 18.7% | 237 | 18.9% | 458 | 26.2% | 950 | 21.8% |
| Very good | 260 | 19.0% | 257 | 20.5% | 444 | 25.4% | 961 | 22.0% |
| Bad or very bad | 388 | 28.4% | 232 | 18.5% | 244 | 14.0% | 864 | 19.8% |

**Table S9f. Conflict with other students or friends – students’ self-report**

If you challenged OTHER STUDENTS OR FRIENDS, how did this make you feel?

|  | Kenya | | Rwanda | | Uganda | | Total | |
| --- | --- | --- | --- | --- | --- | --- | --- | --- |
|  | n | % | n | % | n | % | n | % |
|  | 1367 |  | 1251 |  | 1749 |  | 4367 |  |
| Missing | 4 | 0.3% | 32 | 2.6% | 22 | 1.3% | 58 | 1.3% |
| I did not challenge | 107 | 7.8% | 237 | 18.9% | 237 | 13.6% | 581 | 13.3% |
| Very bad | 103 | 7.5% | 143 | 11.4% | 76 | 4.3% | 322 | 7.4% |
| Bad | 145 | 10.6% | 106 | 8.5% | 73 | 4.2% | 324 | 7.4% |
| Neither bad nor good | 158 | 11.6% | 220 | 17.6% | 267 | 15.3% | 645 | 14.8% |
| Good | 377 | 27.6% | 263 | 21.0% | 447 | 25.6% | 1087 | 24.9% |
| Very good | 473 | 34.6% | 250 | 20.0% | 627 | 35.8% | 1350 | 30.9% |
| Bad or very bad | 248 | 18.1% | 249 | 19.9% | 149 | 8.5% | 646 | 14.8% |

**Table S9g. Stress – students’ self-report**

How stressful were the lessons for you?

|  | Kenya | | Rwanda | | Uganda | | Total | |
| --- | --- | --- | --- | --- | --- | --- | --- | --- |
|  | n | % | n | % | n | % | n | % |
|  | 1367 |  | 1251 |  | 1749 |  | 4367 |  |
| Not stressful at all | 565 | 41.3% | 554 | 44.3% | 632 | 36.1% | 1751 | 40.1% |
| A little stressful | 509 | 37.2% | 391 | 31.3% | 879 | 50.3% | 1779 | 40.7% |
| Stressful | 163 | 11.9% | 147 | 11.8% | 128 | 7.3% | 438 | 10.0% |
| Very stressful | 103 | 7.5% | 129 | 10.3% | 101 | 5.8% | 333 | 7.6% |
| Stressful or very stressful | 266 | 19.5% | 276 | 22.1% | 229 | 13.1% | 771 | 17.7% |

**Table S9h. Other adverse effects – students’ self-report**

Did the lessons have any bad effects or disadvantages for you apart from stress or feeling bad about challenging what others said?

|  | Kenya | | Rwanda | | Uganda | | Total | |
| --- | --- | --- | --- | --- | --- | --- | --- | --- |
|  | n | % | n | % | n | % | n | % |
|  | 1367 |  | 1251 |  | 1749 |  | 4367 |  |
| Missing | 28 | 2.0% | 30 | 2.4% | 5 | 0.3% | 63 | 1.4% |
| Yes | 324 | 23.7% | 412 | 32.9% | 407 | 23.3% | 1143 | 26.2% |
| No | 1,015 | 74.3% | 809 | 64.7% | 1337 | 76.4% | 3161 | 72.4% |

# Tables S10a to S10i. Transfer of what was learned to daily life

**Identification and assessment of claims about the effects of health actions**

**Table S10a. Health action correctly identified**

|  | **Control schools** | **Intervention schools** | **Adjusted difference** | **Odds ratio** | **P** | **ICC** | |
| --- | --- | --- | --- | --- | --- | --- | --- |
|  | N (%) | N (%) |  |  |  | Trial | School |
| Kenya | 272/379 (71.8%) | 311/391 (79.5%) | 7.5% (-0.8 to 15.7) | 1.6 (1.0 to 2.6) | 0.077 |  | 0.16 |
| Rwanda | 269/472 (57.0%) | 260/448 (58.0%) | 1.2% (-9.3 to 11.7) | 1.1 (0.6 to 1.8) | 0.823 |  | 0.23 |
| Uganda | 240/400 (40%) | 302/395 (76.5%) | 16.5% (7.7 to 25.4) | 2.4 (1.5 to 3.8) | <0.001 |  | 0.15 |
| IPD meta-analysis | 781/1251 (62.4%) | 873/1234 (70.8%) | 8.4% (2.8 to 13.9) | 1.6 (1.2 to 2.1) | 0.003 | 0.03 | 0.22 |

**Table S10b. Claimed effect correctly identified**

|  | **Control schools** | **Intervention schools** | **Adjusted difference** | **Odds ratio** | **P** | **ICC** | |
| --- | --- | --- | --- | --- | --- | --- | --- |
|  | N (%) | N (%) |  |  |  | Trial | School |
| Kenya | 250/377 (66.3%) | 284/389 (73.0%) | 6.9% (-1.8 to 15.5) | 1.4 (0.9 to 2.3) | 0.121 |  | 0.13 |
| Rwanda | 252/472 (53.4%) | 224/448 (50.0%) | -3.2% (-13.5 to 7.0) | 0.9 (0.5 to 1.4) | 0.534 |  | 0.20 |
| Uganda | 223/400 (55.8%) | 273/395 (69.1%) | 13.3% (2.8 to 13.9) | 2.0 (1.1 to 3.5) | 0.014 |  | 0.24 |
| IPD meta-analysis | 725/1249 (58.1%) | 781/1232 (63.4%) | 5.6% (-0.2 to 11.4) | 1.3 (1.0 to 1.8) | 0.060 | 0.03 | 0.22 |

**Table S10c. Basis for the claim correctly identified**

|  | **Control schools** | **Intervention schools** | **Adjusted difference** | **Odds ratio** | **P** | **ICC** | |
| --- | --- | --- | --- | --- | --- | --- | --- |
|  | N (%) | N (%) |  |  |  | Trial | School |
| Kenya | 180/307 (58.6%) | 201/306 (65.7%) | 6.6% (-4.4 to 17.5) | 1.4 (0.8 to 2.4) | 0.243 |  | 0.20 |
| Rwanda | 101/210 (48.1%) | 124/198 (62.6%) | 13.3% (1.5 to 25.1) | 1.8 (1.1 to 3.1) | 0.029 |  | 0.14 |
| Uganda | 89/180 (49.4% | 137/250 (54.8%) | 4.9% (-8-0 to 17.7) | 1.3 (0.7 to 2.3) | 0.459 |  | 0.22 |
| IPD meta-analysis | 370/697 (53.1%) | 462/754 (61.3%) | 7.9% (1.0 to 14.8) | 1.5 (1.0 to 2.0) | 0.025 | 0.01 | 0.20 |

**Table S10d. Reliability of the claim correctly assessed**

|  | **Control schools** | **Intervention schools** | **Adjusted difference** | **Odds ratio** | **P** | **ICC** | |
| --- | --- | --- | --- | --- | --- | --- | --- |
|  | N (%) | N (%) |  |  |  | Trial | School |
| Kenya | 68/190 (35.8%) | 108/208 (51.9%) | 16.0% (5.9 to 26.1) | 1.9 (1.3 to 3.0) | 0.002 |  | 0.02 |
| Rwanda | 22/100 (22.0%) | 62/122 (50.8%) | 28.6% (15.6 to 41.6) | 3.9 (2.0 to 7.9) | <0.001 |  | 0.07 |
| Uganda | 65/81 (80.3%) | 115/133 (86.5%) | 4.7% (-6.7 to 16.0) | 1.4 (0.6 to 3.5) | 0.409 |  | 0.13 |
| IPD meta-analysis | 155/371 (41.8%) | 285/463 (61.6%) | 16.1% (8.5 to 23.7) | 2.3 (1.6 to 3.2) | <0.001 | 0.22 | 0.27 |

**Reasons for deciding whether to take the health action**

**Table S10e. Reliability of the claim considered**

|  | **Control schools** | **Intervention schools** | **Adjusted difference** | **Odds ratio** | **P** | **ICC** | |
| --- | --- | --- | --- | --- | --- | --- | --- |
|  | N (%) | N (%) |  |  |  | Trial | School |
| Kenya | 64/182 (35.2%) | 102/203 (50.3%) | 14.3% (3.2 to 25.4) | 1.9 (1.1 to 3.0) | 0.013 |  | 0.07 |
| Rwanda | 7/100 (7.0%) | 34/122 (27.9%) | 20.4% (10.2 to 30.7) | 5.3 (2.1 to 13.7) | <0.001 |  | 0.09 |
| Uganda | 53/82 (64.6%) | 58/135 (43%) | -21.1% (-38.1 to -4.1) | 0.3 (0.1 to 0.8) | 0.019 |  | 0.30 |
| IPD meta-analysis | 214/364 (34.1%) | 194/460 (42.2%) | 6.5% (-1.3 to 14.4) | 1.4 (0.9 to 2.2) | 0.100 | 0.14 | 0.30 |

**Table S10f. Advantages considered**

|  | **Control schools** | **Intervention schools** | **Adjusted difference** | **Odds ratio** | **P** | **ICC** | |
| --- | --- | --- | --- | --- | --- | --- | --- |
|  | N (%) | N (%) |  |  |  | Trial | School |
| Kenya | 85/186 (45.7%) | 86/202 (42.6%) | -4.1% (-16.1 to 7.9) | 0.8 (0.5 to 1.4) | 0.502 |  | 0.13 |
| Rwanda | 8/100 (8.0%) | 5/122 (4.1%) | -3.9% (-10.5 to 2.7) | 0.5 (0.2 to 1.4) | 0.245 |  | 0.08 |
| Uganda | 43/73 (58.9%) | 53/106 (50%) | -11.4 (-29.1 to 6.3) | 0.5 (0.2 to 1.4) | 0.219 |  | 0.24 |
| IPD meta-analysis | 136/359 (37.9%) | 144/430 (33.5%) | -5.4% (-12.5 to 1.6) | 0.7 (0.5 to 1.1) | 0.117 | 0.33 | 0.44 |

**Table S10g. Disadvantages considered**

|  | **Control schools** | **Intervention schools** | **Adjusted difference** | **Odds ratio** | **P** | **ICC** | |
| --- | --- | --- | --- | --- | --- | --- | --- |
|  | N (%) | N (%) |  |  |  | Trial | School |
| Kenya | 75/186 (40.3%) | 104/203 (51.2%) | 12.0% (-0.3 to 24.4) | 1.7 (1.0 to 3.1) | 0.061 |  | 0.17 |
| Rwanda | 10/100 (10.0%) | 14/122 (11.5%) | 1.7 % (-7.2 to 10.6) | 1.2 (0.4 to 3.3) | 0.710 |  | 0.17 |
| Uganda | 25/74 (33.8%) | 50/121 (41.3%) | 7.3% (-11.1 to 25.8) | 1.6 (0.5 to 5.5) | 0.443 |  | 0.48 |
| IPD meta-analysis | 110/360 (30.6%) | 168/446 (37.7%) | 7.3% (-0.5 to 15.1) | 1.6 (1.0 to 2.6) | 0.060 | 0.17 | 0.38 |

**Table S10h. Personal experience considered**

|  | **Control schools** | **Intervention schools** | **Adjusted difference** | **Odds ratio** | **P** | **ICC** | |
| --- | --- | --- | --- | --- | --- | --- | --- |
|  | N (%) | N (%) |  |  |  | Trial | School |
| Kenya | 177/188 (94.2%) | 188/204 (92.2%) | -1.8% (-7.2 to 3.6) | 0.8 (0.3 to 1.8) | 0.521 |  | 0.10 |
| Rwanda | 48/60 (80%) | 50/80 (62.5%) | -15.6% (-32.4 to 1.2) | 0.4 (0.2 to 1.1) | 0.076 |  | 0.18 |
| Uganda | 40/45 (88.9%) | 41/50 (82.0%) | -6.9% (-22.0 to 8.1) | 0.6 (0.1 to 2.0) | 0.376 |  | 0.11 |
| IPD meta-analysis | 265/293 (90.4%) | 279/334 (83.5%) | -6.9% (-14.3 to 0.7) | 0.6 (0.3 to 1.0) | 0.056 | 0.12 | 0.26 |

**Table S10i. Health professional or researcher advice considered**

|  | **Control schools** | **Intervention schools** | **Adjusted difference** | **Odds ratio** | **P** | **ICC** | |
| --- | --- | --- | --- | --- | --- | --- | --- |
|  | N (%) | N (%) |  |  |  | Trial | School |
| Kenya | 8/188 (4.3%) | 12/204 (5.9%) | 1.5% (-3.2 to 6.2) | 1.4 (0.5 to 3.8) | 0.541 |  | 0.14 |
| Rwanda | 12/60 (20.0%) | 27/80 (33.7%) | 11.8% (-5.2 to 28.8) | 2.1 (0.7 to 5.9) | 0.183 |  | 0.25 |
| Uganda | 5/45 (11.1%) | 9/50 (18.0%) | 6.9% (-8.1 to 22.0) | 1.8 (o.5 to 6.7) | 0.376 |  | 0.12 |
| IPD meta-analysis | 25/293 (8.5%) | 48/334 (14.4%) | 5.8% (-1.6 to 13.3) | 1.7 (0.9 to 3.3) | 0.101 | 0.15 | 0.32 |

# Tables S11a to S11g. Intervention school teachers – transfer and adverse effects

**Table S11a. Use of what was learned by students – teachers’ observations**

Have you noticed your students using what they learned from the lessons?

|  | Kenya | | Rwanda | | Uganda | | Total | |
| --- | --- | --- | --- | --- | --- | --- | --- | --- |
|  | n | % | n | % | n | % | n | % |
|  | 33 |  | 36 |  | 40 |  | 109 |  |
| Missing | 1 | 3.0% | 0 | 0.0% | 0 | 0.0% | 1 | 0.9% |
| Not at all | 1 | 3.0% | 2 | 5.6% | 3 | 7.5% | 6 | 5.5% |
| Rarely | 2 | 6.1% | 4 | 11.1% | 0 | 0.0% | 6 | 5.5% |
| Sometimes | 19 | 57.6% | 19 | 52.8% | 31 | 77.5% | 69 | 63.3% |
| A lot | 10 | 30.3% | 11 | 30.6% | 6 | 15.0% | 27 | 24.8% |
| Rarely or not at all | 3 | 9.1% | 6 | 16.7% | 3 | 7.5% | 12 | 11.0% |
| Sometimes or a lot | 29 | 87.9% | 30 | 83.3% | 37 | 92.5% | 96 | 88.1% |

**Table S11b. Use of what was learned by teachers – self-report**

How much have you used what you learned from the lessons?

|  | Kenya | | Rwanda | | Uganda | | Total | |
| --- | --- | --- | --- | --- | --- | --- | --- | --- |
|  | n | % | n | % | n | % | n | % |
|  | 33 |  | 36 |  | 40 |  | 109 |  |
| Missing | 1 | 3.0% | 0 | 0.0% | 0 | 0.0% | 1 | 0.9% |
| Not at all | 0 | 0.0% | 3 | 8.3% | 0 | 0.0% | 3 | 2.8% |
| Rarely | 2 | 6.1% | 3 | 8.3% | 0 | 0.0% | 5 | 4.6% |
| Sometimes | 5 | 15.2% | 11 | 30.6% | 15 | 37.5% | 31 | 28.4% |
| A lot | 25 | 75.8% | 19 | 52.8% | 25 | 62.5% | 69 | 63.3% |
| Rarely or not at all | 2 | 6.1% | 6 | 16.7% | 0 | 0.0% | 8 | 7.3% |
| Sometimes or a lot | 30 | 90.9% | 30 | 83.3% | 40 | 100.0% | 100 | 91.7% |

**Table S11c. Use in teaching other subjects – self-report**

Have you used what you learned from preparing and teaching the lessons in teaching other subjects?

|  | Kenya | | Rwanda | | Uganda | | Total | |
| --- | --- | --- | --- | --- | --- | --- | --- | --- |
|  | n | % | n | % | n | % | n | % |
|  | 33 |  | 36 |  | 40 |  | 109 |  |
| Missing | 1 | 3.0% | 0 | 0.0% | 1 | 2.5% | 2 | 1.8% |
| Not at all | 0 | 0.0% | 4 | 11.1% | 1 | 2.5% | 5 | 4.6% |
| Rarely | 0 | 0.0% | 2 | 5.6% | 1 | 2.5% | 3 | 2.8% |
| Sometimes | 16 | 48.5% | 11 | 30.6% | 22 | 55.0% | 49 | 45.0% |
| A lot | 16 | 48.5% | 19 | 52.8% | 15 | 37.5% | 50 | 45.9% |
| Rarely or not at all | 0 | 0.0% | 6 | 16.7% | 2 | 5.0% | 8 | 7.3% |
| Sometimes or a lot | 32 | 97.0% | 30 | 83.3% | 37 | 92.5% | 99 | 90.8% |

**Table S11d. Conflict with teachers – teachers’ observations**

If students challenged things that you said, how did this make you feel?

|  | Kenya | | Rwanda | | Uganda | | Total | |
| --- | --- | --- | --- | --- | --- | --- | --- | --- |
|  | n | % | n | % | n | % | n | % |
|  | 33 |  | 36 |  | 40 |  | 109 |  |
| Missing | 0 | 0.0% | 0 | 0.0% | 0 | 0.0% | 0 | 0.0% |
| They did not challenge things that I said | 2 | 6.1% | 10 | 27.8% | 5 | 12.5% | 17 | 15.6% |
| Very bad | 1 | 3.0% | 3 | 8.3% | 1 | 2.5% | 5 | 4.6% |
| Bad | 1 | 3.0% | 5 | 13.9% | 0 | 0.0% | 6 | 5.5% |
| Neither good nor bad | 1 | 3.0% | 5 | 13.9% | 6 | 15.0% | 12 | 11.0% |
| Good | 20 | 60.6% | 13 | 36.1% | 14 | 35.0% | 47 | 43.1% |
| Very good | 8 | 24.2% | 0 | 0.0% | 14 | 35.0% | 22 | 20.2% |
| Bad or very bad | 2 | 6.1% | 8 | 22.2% | 1 | 2.5% | 11 | 10.1% |

**Table S11e. Stress - teachers**

How stressful was it for you to prepare and teach these lessons?

|  | Kenya | | Rwanda | | Uganda | | Total | |
| --- | --- | --- | --- | --- | --- | --- | --- | --- |
|  | n | % | n | % | n | % | n | % |
|  | 33 |  | 36 |  | 40 |  | 109 |  |
| Missing | 0 | 0.0% | 0 | 0.0% | 0 | 0.0% | 0 | 0.0% |
| Not stressful at all | 16 | 48.5% | 15 | 41.7% | 16 | 40.0% | 47 | 43.1% |
| A little stressful | 16 | 48.5% | 10 | 27.8% | 23 | 57.5% | 49 | 45.0% |
| Stressful | 1 | 3.0% | 10 | 27.8% | 1 | 2.5% | 12 | 11.0% |
| Very stressful | 0 | 0.0% | 1 | 2.8% | 0 | 0.0% | 1 | 0.9% |
| Stressful or very stressful | 1 | 3.0% | 11 | 30.6% | 1 | 2.5% | 13 | 11.9% |

**Table S11f. Reasons for stress - teachers**

Which of the following made the lessons stressful?

|  | Kenya | | Rwanda | | Uganda | | Total | |
| --- | --- | --- | --- | --- | --- | --- | --- | --- |
|  | n | % | n | % | n | % | n | % |
|  | 33 |  | 36 |  | 40 |  | 109 |  |
| Taking time to prepare for the lessons | 5 | 15.2% | 2 | 5.6% | 9 | 22.5% | 16 | 14.7% |
| Taking time away from other subjects | 11 | 33.3% | 8 | 22.2% | 8 | 20.0% | 27 | 24.8% |
| Completing the lessons during the time available | 11 | 33.3% | 12 | 33.3% | 9 | 22.5% | 32 | 29.4% |
| Having to learn new material or methods | 2 | 6.1% | 3 | 8.3% | 4 | 10.0% | 9 | 8.3% |
| Getting off track because of students asking questions | 4 | 12.1% | 0 | 0.0% | 8 | 20.0% | 12 | 11.0% |
| Feeling unprepared to answer students’ questions | 1 | 3.0% | 1 | 2.8% | 0 | 0.0% | 2 | 1.8% |
| Having students challenge things that I said | 0 | 0.0% | 1 | 2.8% | 0 | 0.0% | 1 | 0.9% |
| Teaching students who lacked motivation | 1 | 3.0% | 0 | 0.0% | 3 | 7.5% | 4 | 3.7% |

**Table S11g. Students’ misunderstandings – teachers’ observations**

Have you observed any of your students relying on any of the unreliable claims that were used as examples in the lessons?

|  | Kenya | | Rwanda | | Uganda | | Total | |
| --- | --- | --- | --- | --- | --- | --- | --- | --- |
|  | n | % | n | % | n | % | n | % |
|  | 33 |  | 36 |  | 40 |  | 109 |  |
| Missing | 0 | 0.0% | 0 | 0.0% | 0 | 0.0% | 0 | 0.0% |
| Not at all | 9 | 27.3% | 7 | 19.4% | 7 | 17.5% | 23 | 21.1% |
| Rarely | 11 | 33.3% | 9 | 25.0% | 12 | 30.0% | 32 | 29.4% |
| Sometimes | 12 | 36.4% | 14 | 38.9% | 17 | 42.5% | 43 | 39.4% |
| A lot | 1 | 3.0% | 6 | 16.7% | 4 | 10.0% | 11 | 10.1% |
| Sometimes or a lot | 13 | 39.4% | 20 | 55.6% | 21 | 52.5% | 54 | 49.5% |

# Table S12a to S12d. Potential effect modifiers

**Table S12a. Potential effect modifiers - all students**

|  | **One year** | | **Just after the intervention** | |
| --- | --- | --- | --- | --- |
| **Factor (comparison)** | **Interaction between intervention and factor** | **𝘱-value** | **Interaction between intervention and factor** | **𝘱-value** |
| Outcome | (95% confidence interval) |  | (95% confidence interval) |  |
| **Version (blackboard* vs projector)** |  |  |  |  |
| Passing score (≥ 9 out of 18 correct answers) ^†^ | 1.79 (0.59 to 5.45) | 0.303 | 1.71 (1.02 to 2.86) | 0.040 |
| Mastery score (≥ 14 out of 18 correct answers)^†^ | 0.69 (0.35 to 1.38) | 0.296 | 2.39 (1.00 to 5.70) | 0.051 |
| Test score (% correct)³ | 25.9% (-11.1% to 63.0%) | 0.170 | 26.3% (1.3% to 51.2%) | 0.039 |
| **Class size (continuous variable)^‡^** |  |  |  |  |
| Passing score (≥ 9 out of 18 correct answers)^†^ | 0.99 (0.98 to 0.99) | 0.002 | 0.99 (0.98 to 1.00) | 0.102 |
| Mastery score (≥ 14 out of 18 correct answers)^†^ | 0.99 (0.98 to 1.01) | 0.262 | 1.00 (0.98 to 1.02) | 0.993 |
| Test score (% correct)^‡^ | -0.5% (-0.9% to -0.0%) | 0.031 | -0.2% (-0.8% to 0.4%) | 0.487 |
| **Performance (low* versus moderate or high)** |  |  |  |  |
| Passing score (≥ 9 out of 18 correct answers)^†^ | 1.22 (0.95 to 1.57) | 0.126 | 1.57 (1.15 to 2.16) | 0.005 |
| Mastery score (≥ 14 out of 18 correct answers)^†^ | 1.34 (0.54 to 3.34) | 0.525 | 1.55 (0.74 to 3.23) | 0.247 |
| Test score (% correct)^‡^ | 32.8% (20.8% to 44.8%) | <0.0001 | 42.3% (29.0% to 55.6%) | <0.0001 |
| **Gender (female* versus male)** |  |  |  |  |
| Passing score (≥ 9 out of 18 correct answers)^†^ | 1.33 (1.13 to 1.57) | 0.001 | 1.49 (1.23 to 1.81) | <0.0001 |
| Mastery score (≥ 14 out of 18 correct answers)^†^ | 0.93 (0.60 to 1.43) | 0.733 | 1.87 (1.05 to 3.33) | 0.034 |
| Test score (% correct)^§^ | 8.7% (0.2% to 17.1%) | 0.044 | 10.2% (2.8% to 17.5%) | 0.007 |

* Reference group.

^†^ Estimates of interactions are odds ratios adjusted for projector use, class size, performance, gender, and school ownership (public or government-funded versus private), and the interaction terms. Odds ratios greater than one suggest the intervention (rather than the control) is associated with higher odds of passing or mastery in the comparison group compared to the reference group.

^‡^ The unit of measurement for class size was student (e.g., increasing an intervention class size by 10 students would be expected to decrease the odds of passing with an odds ratio of 0.86; 95% CI 0.78 to 0.94).). Hierarchical random intercepts were used to model clustering of students within schools, and schools within country (trial).

^§^ Estimates of interactions are differences in mean test scores, expressed as percentages, and adjusted in the same way as for the dichotomous outcomes. Differences greater than zero suggest the intervention (rather than the control) is associated with higher scores in the comparison group compared to the reference group.

**Table S12b. Potential effect modifiers – Kenyan trial**

| **Factor (comparison)** | **Interaction between intervention and factor** | **𝘱-value** |
| --- | --- | --- |
| Outcome | (95% confidence interval) |  |
| **Class size (continuous variable)*** |  |  |
| Passing score (≥ 9 out of 18 correct answers)^†^ | 0.99 (0.97 to 1.01) | 0.457 |
| Mastery score (≥ 14 out of 18 correct answers)^†^ | 1.02 (0.98 to 1.06) | 0.307 |
| Test score (% correct)^‡^ | -0.0% (-1.0% to 0.9%) | 0.969 |
| **Performance (low^§^ versus moderate or high)** |  |  |
| Passing score (≥ 9 out of 18 correct answers)^†^ | 1.40 (0.85 to 2.30) | 0.186 |
| Mastery score (≥ 14 out of 18 correct answers)^†^ | 0.30 (0.04 to 2.31) | 0.247 |
| Test score (% correct)^‡^ | 36.2% (15.1% to 57.2%) | 0.001 |
| **Gender (female^§^ versus male)** |  |  |
| Passing score (≥ 9 out of 18 correct answers)^†^ | 1.66 (1.02 to 2.70) | 0.042 |
| Mastery score (≥ 14 out of 18 correct answers)^†^ | 0.81 (0.29 to 2.25) | 0.681 |
| Test score (% correct)^‡^ | 21.2% (2.7% to 39.7%) | 0.025 |

* The unit of measurement for class size was student (e.g., increasing an intervention class size by 10 students would be expected to decrease the odds of passing with an odds ratio of 0.92; 95% CI 0.74 to 1.15). Hierarchical random intercepts were used to model clustering of students within schools, and schools within country (trial).

^†^ Estimates of interactions are odds ratios adjusted for projector use, class size, performance, gender, and school ownership (public or government-funded versus private), and the interaction terms. Odds ratios greater than one suggest the intervention (rather than the control) is associated with higher odds of passing or mastery in the comparison group compared to the reference group.

^‡^ Estimates of interactions are differences in mean test scores, expressed as percentages, and adjusted in the same way as for the dichotomous outcomes. Differences greater than zero suggest the intervention (rather than the control) is associated with higher scores in the comparison group compared to the reference group.

^§^ Reference group.

**Table S12c. Potential effect modifiers – Rwandan trial**

| **Factor (comparison)** | **Interaction between intervention and factor** | **𝘱-value** |
| --- | --- | --- |
| Outcome | (95% confidence interval) |  |
| **Class size (continuous variable)*** |  |  |
| Passing score (≥ 9 out of 18 correct answers)^†^ | 0.98 (0.93 to 1.03) | 0.387 |
| Mastery score (≥ 14 out of 18 correct answers)^†^ | 0.97 (0.90 to 1.04) | 0.405 |
| Test score (% correct)^‡^ | -0.4% (-2.5% to 1.8%) | 0.737 |
| **Performance (low^§^ versus moderate or high)** |  |  |
| Passing score (≥ 9 out of 18 correct answers)^†^ | 1.00 (0.35 to 2.84) | 0.999 |
| Mastery score (≥ 14 out of 18 correct answers)^†^ | 2.29 (0.51 to 10.31) | 0.281 |
| Test score (% correct)^‡^ | 55.0% (8.5% to 101.6%) | 0.020 |
| **Gender (female^§^ versus male)** |  |  |
| Passing score (≥ 9 out of 18 correct answers)^†^ | 1.22 (0.77 to 1.92) | 0.399 |
| Mastery score (≥ 14 out of 18 correct answers)^†^ | 0.56 (0.23 to 1.36) | 0.197 |
| Test score (% correct)^‡^ | 6.6% (-10.8% to 23.9%) | 0.459 |

* The unit of measurement for class size was student (e.g., increasing an intervention class size by 10 students would be expected to decrease the odds of passing with an odds ratio of 0.81; 95% CI 0.51 to 1.30). Hierarchical random intercepts were used to model clustering of students within schools, and schools within country (trial).

^†^ Estimates of interactions are odds ratios adjusted for projector use, class size, performance, gender, and school ownership (public or government-funded versus private), and the interaction terms. Odds ratios greater than one suggest the intervention (rather than the control) is associated with higher odds of passing or mastery in the comparison group compared to the reference group.

^‡^ Estimates of interactions are differences in mean test scores, expressed as percentages, and adjusted in the same way as for the dichotomous outcomes. Differences greater than zero suggest the intervention (rather than the control) is associated with higher scores in the comparison group compared to the reference group.

^§^ Reference group.

**Table S12d. Potential effect modifiers – Ugandan trial**

| **Factor (comparison)** | **Interaction between intervention and factor** | **𝘱-value** |
| --- | --- | --- |
| Outcome | (95% confidence interval) |  |
| **Class size (continuous variable)*** |  |  |
| Passing score (≥ 9 out of 18 correct answers)^†^ | 0.82 (0.45 to 1.52) | 0.537 |
| Mastery score (≥ 14 out of 18 correct answers)^†^ | 0.54 (0.18 to 1.65) | 0.281 |
| Test score (% correct)^‡^ | 6.3% (-32.8% to 45.4%) | 0.752 |
| **Performance (low^§^ versus moderate or high)** |  |  |
| Passing score (≥ 9 out of 18 correct answers)^†^ | 0.99 (0.98 to 1.00) | 0.096 |
| Mastery score (≥ 14 out of 18 correct answers)^†^ | 1.00 (0.98 to 1.02) | 0.819 |
| Test score (% correct)^‡^ | -0.5% (-1.1% to 0.1%) | 0.084 |
| **Gender (female^§^ versus male)** |  |  |
| Passing score (≥ 9 out of 18 correct answers)^†^ | 0.94 (0.55 to 1.58) | 0.803 |
| Mastery score (≥ 14 out of 18 correct answers)^†^ | 1.58 (0.59 to 4.26) | 0.367 |
| Test score (% correct)^‡^ | 2.3% (-29.7% to 34.3%) | 0.888 |

* The unit of measurement for class size was student (e.g., increasing an intervention class size by 10 students would be expected to decrease the odds of passing with an odds ratio of 0.91; 95% CI 0.81 to 1.02). Hierarchical random intercepts were used to model clustering of students within schools, and schools within country (trial).

^†^ Estimates of interactions are odds ratios adjusted for projector use, class size, performance, gender, and school ownership (public or government-funded versus private), and the interaction terms. Odds ratios greater than one suggest the intervention (rather than the control) is associated with higher odds of passing or mastery in the comparison group compared to the reference group.

^‡^ Estimates of interactions are differences in mean test scores, expressed as percentages, and adjusted in the same way as for the dichotomous outcomes. Differences greater than zero suggest the intervention (rather than the control) is associated with higher scores in the comparison group compared to the reference group.

^§^ Reference group.

# Table S13. Credibility of the effect modifier analyses for the primary outcome

| **Criteria*** | | **Potential effect modifiers^†^** | | | |
| --- | --- | --- | --- | --- | --- |
|  | Version | | Performance | Class size | Sex |
| 1: Is the analysis of effect modification based on comparison within rather than between trials? | No | | Yes | Yes | Yes |
| 2: For within-trial comparisons, is the effect modification similar from trial to trial? | - | | Yes | Yes | Yes |
| 3: For between-trial comparisons, is the number of trials large? | No | | NA | NA | NA |
| 4: Was the direction of the effect modification correctly hypothesized a priori? | Yes | | Yes | Yes | No |
| 5: Does a test for interaction suggest that chance is an unlikely explanation of the apparent effect modification? | No | | No | Yes | Yes |
| 6: Did the authors test only a small number of effect modifiers or consider the number in their statistical analysis? | Yes | | Yes | Yes | Yes |
| 7: Did the authors use a random effects model? | Yes | | Yes | Yes | Yes |
| 8: If the effect modifier is a continuous variable, were arbitrary cut points avoided? | NA | | NA | Yes | NA |
| 9 Optional: Are there any additional considerations that may increase or decrease credibility? | - | | - | - | - |
| 10: How would you rate the overall credibility of the proposed effect modification? | Very low‡ | | Low^§^ | High | Moderate** |

* Instrument for assessing the Credibility of Effect Modification Analyses (ICEMAN) criteria for meta-analyses of randomized controlled trials.

Schandelmaier S, Briel M, Varadhan R, et al. Development of the Instrument to assess the Credibility of Effect Modification Analyses (ICEMAN) in randomized controlled trials and meta-analyses. *CMAJ* 2020; **192**(32): E901-e6.

† Version = use of projector vs blackboard versions of the lessons. Performance = low vs moderate or high performance on end-of-term examinations for the previous school term. Class size = number of students in the class where the lessons were taught. Sex = female vs male.

‡ Version: A within country comparison was only possible in the Ugandan trial (Table S9d), where there was not evidence of an interaction. There was not evidence of an interaction in the IPD meta-analysis (Table S9a) or in the Ugandan trial (Table S9d). None of the schools in Kenya used the projector version, all the schools in Rwanda used the projector version, and stratified random allocation was used to ensure a fair distribution of schools with and without a projector in Uganda. There were only three trials and there may be confounding due to other differences between the Kenyan and Rwandan trials.

^§^ Performance: The evidence of an interaction is based on just one of the three trials. Performance was measured at the level of student in Kenya (Table S9b) where the odds ratio for an interaction was 1.40 (95% CI 0.85-2.30, p=0.186). The odds ratio for an interaction in the IPD meta-analysis (Table S9a) was 1.22 (95% CI 0.95-1.57, p=0.126). Performance was measured at the level of school in Rwanda and Uganda where was not evidence of an interaction (Tables S9c and S9d).

** This analysis was not specified in the protocol, and we did not have an a priori hypothesis.

# Table S14a to 14c. English reading proficiency subgroup analyses

**Table S14a. English reading proficiency subgroup analysis – passing score**

|  | **Control** | **Intervention** | **Adjusted difference** | **Odds ratio** | **P value** | **ICC** | |
| --- | --- | --- | --- | --- | --- | --- | --- |
|  |  |  |  |  |  | **Trial** | **School** |
| **Advanced Proficiency** |  |  |  |  |  |  |  |
|  | 3790 students | 4381 students |  |  |  |  |  |
|  | 122 schools | 122 schools |  |  |  |  |  |
| Students with a passing score (≥9/18) | 743／2278 (32.6%) | 1630／2794 (58.3%) | 27.0% (15.1%–38.9%) | 3.7 (1.9–7.4) | 0.0002 | <0.001 | 0.151 |
| **Basic Proficiency** |  |  |  |  |  |  |  |
|  | 3333 students | 3706 students |  |  |  |  |  |
|  | 122 schools | 122 schools |  |  |  |  |  |
| Students with a passing score (≥9/18) | 556／1821 (30.5%) | 1135／2119 (53.6%) | 24.8% (13.0%–36.7%) | 3.5 (1.6–7.5) | 0.0014 | <0.001 | 0.160 |
| **Lacking Proficiency** |  |  |  |  |  |  |  |
|  | 3437 students | 3672 students |  |  |  |  |  |
|  | 122 schools | 122 schools |  |  |  |  |  |
| Students with a passing score (≥9/18) | 496／1925 (25.8%) | 936／2085 (44.9%) | 14.9% (11.2%–18.7%) | 2.4 (1.6–3.5) | <0.0001 | 0.078 | 0.201 |
| **Interactions with Reading Proficiency** | |  |  |  |  |  |  |
| Intervention × Basic Proficiency | |  |  | 0.8 (0.7–0.8) | <0.0001 |  |  |
| Intervention × Lacking Proficiency | |  |  | 0.4 (0.3–0.7) | 0.0004 |  |  |
| Joint test of no interaction | |  |  |  | <0.0001 |  |  |

English reading skills were assessed using four questions at the beginning of the CTH test: two advanced and two basic questions. We categorized students’ reading skills as “advanced” (all four questions correct), “basic” (both basic questions correct and one or both advanced questions wrong), and “lacking basic” (one or both basic questions wrong).

Data are n／N (%) or % (95% CI). Clustering was accounted for using random intercepts at the level of trial and school within trial. Logistic regression was used to estimate adjusted odds ratios, which are re-expressed as adjusted differences. Intraclass correlation coefficients (ICCs) are estimated at the same levels as the random intercepts (trial and school within trial). Fixed effects were used to adjust all estimates as described in the Methods section. Wald-type confidence intervals and two-sided p-values were computed in all analyses. Advanced English reading proficiency was used as the reference.

**Table S14b. English reading proficiency subgroup analysis – mastery**

|  | **Control** | **Intervention** | **Adjusted difference** | **Odds ratio** | **P value** | **ICC** | |
| --- | --- | --- | --- | --- | --- | --- | --- |
|  |  |  |  |  |  | **Trial** | **School** |
| **Advanced Proficiency** |  |  |  |  |  |  |  |
|  | 3790 students | 4381 students |  |  |  |  |  |
|  | 122 schools | 122 schools |  |  |  |  |  |
| Students with a passing score (≥9/18) | 67／2278 (2.9%) | 553／2794 (19.8%) | 16.6% (10.4%–22.8%) | 9.2 (7.2–11.7) | <0.0001 | 0.039 | 0.267 |
| **Basic Proficiency** |  |  |  |  |  |  |  |
|  | 3333 students | 3706 students |  |  |  |  |  |
|  | 122 schools | 122 schools |  |  |  |  |  |
| Students with a passing score (≥9/18) | 30／1821 (1.6%) | 261／2119 (12.3%) | 10.5% (7.3%–13.7%) | 10.2 (5.0–20.6) | <0.0001 | <0.001 | 0.246 |
| **Lacking Proficiency** |  |  |  |  |  |  |  |
|  | 3437 students | 3672 students |  |  |  |  |  |
|  | 122 schools | 122 schools |  |  |  |  |  |
| Students with a passing score (≥9/18) | 32／1925 (1.7%) | 192／2085 (9.2%) | 5.0% (-0.1%–10.1%) | 4.7 (1.9–11.8) | 0.0009 | 0.047 | 0.293 |
| **Interactions with Reading Proficiency** | |  |  |  |  |  |  |
| Intervention × Basic Proficiency | |  |  | 0.9 (0.6–1.5) | 0.8165 |  |  |
| Intervention × Lacking Proficiency | |  |  | 0.3 (0.1–0.7) | 0.0038 |  |  |
| Joint test of no interaction | |  |  |  | <0.0001 |  |  |

English reading skills were assessed using four questions at the beginning of the CTH test: two advanced and two basic questions. We categorized students’ reading skills as “advanced” (all four questions correct), “basic” (both basic questions correct and one or both advanced questions wrong), and “lacking basic” (one or both basic questions wrong).

Data are n／N (%) or % (95% CI). Clustering was accounted for using random intercepts at the level of trial and school within trial. Logistic regression was used to estimate adjusted odds ratios, which are re-expressed as adjusted differences. Intraclass correlation coefficients (ICCs) are estimated at the same levels as the random intercepts (trial and school within trial). Fixed effects were used to adjust all estimates as described in the Methods section. Wald-type confidence intervals and two-sided p-values were computed in all analyses. Advanced English reading proficiency was used as the reference.

**Table S14c. English reading proficiency subgroup analysis – mean score**

|  | **Control** | **Intervention** | **Adjusted difference** | **Odds ratio** | **P value** | **ICC** | |
| --- | --- | --- | --- | --- | --- | --- | --- |
|  |  |  |  |  |  | **Trial** | **School** |
| **Advanced Proficiency** |  |  |  |  |  |  |  |
|  | 3790 students | 4381 students |  |  |  |  | 3790 students |
|  | 122 schools | 122 schools |  |  |  |  | 122 schools |
| Students with a passing score (≥9/18) | 40.7% (17.0%) | 53.7% (21.8%) | 13.5% (6.7%–20.4%) | 0.0001 | 0.006 | 0.177 | 40.7% (17.0%) |
| **Basic Proficiency** |  |  |  |  |  |  |  |
|  | 3333 students | 3706 students |  |  |  |  | 3333 students |
|  | 122 schools | 122 schools |  |  |  |  | 122 schools |
| Students with a passing score (≥9/18) | 39.4% (16.1%) | 49.9% (19.7%) | 11.7% (6.8%–16.6%) | <0.0001 | <0.001 | 0.176 | 39.4% (16.1%) |
| **Lacking Proficiency** |  |  |  |  |  |  |  |
|  | 3437 students | 3672 students |  |  |  |  | 3437 students |
|  | 122 schools | 122 schools |  |  |  |  | 122 schools |
| Students with a passing score (≥9/18) | 37.5% (16.1%) | 46.1% (19.1%) | 7.1% (4.9%–9.3%) | <0.0001 | 0.046 | 0.167 | 37.5% (16.1%) |
| **Interactions with Reading Proficiency** | |  |  |  |  |  |  |
| Intervention × Basic Proficiency | |  |  | -3.3% (-4.1%–-2.4%) | <0.0001 |  |  |
| Intervention × Lacking Proficiency | |  |  | -8.8% (-12.2%–-5.4%) | <0.0001 |  |  |
| Joint test of no interaction | |  |  |  | <0.0001 |  |  |

English reading skills were assessed using four questions at the beginning of the CTH test: two advanced and two basic questions. We categorized students’ reading skills as “advanced” (all four questions correct), “basic” (both basic questions correct and one or both advanced questions wrong), and “lacking basic” (one or both basic questions wrong).

Data are % (SD). Clustering was accounted for using random intercepts at the level of trial and school within trial. Linear regression was used to estimate adjusted differences in mean scores. Intraclass correlation coefficients (ICCs) are estimated at the same levels as the random intercepts (trial and school within trial). Fixed effects were used to adjust all estimates as described in the Methods section. Wald-type confidence intervals and two-sided p-values were computed in all analyses. Advanced English reading proficiency was used as the reference.

# Table S15. Risk of bias assessment

| **Studies** | **Randomization process** | **Timing of identification or recruitment of participants** | **Deviations from intended interventions** | **Missing outcome data** | **Measurement of the outcome** | **Selection of the reported result** | **Overall, Bias** |
| --- | --- | --- | --- | --- | --- | --- | --- |
| Students with a passing score | | | | | | | |
| Kenya | Low | Low | Low | Moderate | Low | Low | Moderate |
| Uganda | Low | Low | Low | Moderate | Low | Low | Moderate |
| Rwanda | Low | Low | Low | Moderate | Low | Low | Moderate |
| Students with a mastery score | | | | | | | |
| Kenya | Low | Low | Low | Moderate | Low | Low | Moderate |
| Uganda | Low | Low | Low | Moderate | Low | Low | Moderate |
| Rwanda | Low | Low | Low | Moderate | Low | Low | Moderate |
| Students' mean score | | | | | | | |
| Kenya | Low | Low | Low | Moderate | Low | Low | Moderate |
| Uganda | Low | Low | Low | Moderate | Low | Low | Moderate |
| Rwanda | Low | Low | Low | Moderate | Low | Low | Moderate |
| Teachers with a passing score | | | | | | | |
| Kenya | Low | Low | Low | Moderate | Low | Low | Moderate |
| Uganda | Low | Low | Low | Moderate | Low | Low | Moderate |
| Rwanda | Low | Low | Low | Moderate | Low | Low | Moderate |
| Teachers with a mastery score | | | | | | | |
| Kenya | Low | Low | Low | Moderate | Low | Low | Moderate |
| Uganda | Low | Low | Low | Moderate | Low | Low | Moderate |
| Rwanda | Low | Low | Low | Moderate | Low | Low | Moderate |
| Teachers' mean score | | | | | | | |
| Kenya | Low | Low | Low | Moderate | Low | Low | Moderate |
| Uganda | Low | Low | Low | Moderate | Low | Low | Moderate |
| Rwanda | Low | Low | Low | Moderate | Low | Low | Moderate |

Initial assessments (for results just after the intervention) were made by two independent judges (Xiajing Chu and Liam Yao) using the Cochrane Risk of Bias tool (RoB 2 for cluster-randomized trials)^1,2^ for the following outcomes: passing scores (≥ 9 of 18 correct answers), mastery scores (≥ 14 of 18 correct answers), and mean scores for both students and teachers. The only change for the one-year follow-up results is loss to follow-up, which was 27.9% and 26.5% for students in the control and intervention schools respectively, and 14.8% and 11.5% for teachers in the control and intervention schools respectively. They modified the assessments of missing outcome data and overall bias from low to moderate for this reason.

**References**

1. Eldridge S, Campbell M, Campbell M, et al. Revised Cochrane risk of bias tool for randomized trials (RoB 2): Additional considerations for cluster-randomized trials (RoB 2 CRT). 2021. https://www.riskofbias.info/welcome/rob-2-0-tool/rob-2-for-cluster-randomized-trials.

2. Sterne JAC, Savović J, Page MJ, et al. RoB 2: a revised tool for assessing risk of bias in randomized trials. BMJ 2019; 366: l4898.

# Table S16. GRADE evidence profile

**Population**: lower secondary school students (age 14-16) and their teachers

**Intervention**: IHC secondary school intervention

**Comparator**: Standard national curriculum

| **Outcome**  Timeframe | **Study results and measurements*** | **Absolute effect estimates** | | **Certainty of the Evidence^†^**  (Quality of evidence) | **Plain language summary** |
| --- | --- | --- | --- | --- | --- |
|  |  | Standard national curriculum* | IHC secondary school intervention* |  |  |
| Students with a passing score  (≥ 9 of 18 correct answers) | Odds ratio: 3.7  (2.9-4.6)  Based on data from total sample size 8298 (effective sample size 1324)^‡^ in 3 studies | **287**  per 1000 | **598**  per 1000 | **Moderate**  Due to missing outcome data^§^ | The IHC secondary school intervention probably increase the proportion of students with a passing score by at least 20% after one year. |
|  |  | Difference: **311 more per 1000**  (CI 95% 252-362 more) | |  |  |
| Students with a mastery score  (≥ 14 of 18 correct answers) | Odds ratio: 8.1  (CI 95% 5.6-11.8)  Based on data from total sample size 8298 (effective sample size 974)^‡^ in 3 studies | **24**  per 1000 | **165**  per 1000 | **Moderate**  Due to missing outcome data^§^ | The IHC secondary school intervention probably increases the proportion of students with a mastery score after one year. |
|  |  | Difference: **141 more per 1000**  (CI 95% 96-199 more) | |  |  |
| Students' mean score | Based on data from total sample size 8298 (effective sample size 1276)^‡^ in 3 studies | **38.8**  Mean | **50.1**  Mean | **Moderate**  Due to missing outcome data^§^ | The IHC secondary school intervention probably improves students' mean score after one year. |
|  |  | Difference: **MD 12.3 higher**  (CI 95% 10.2-14.4 higher) | |  |  |
| Teachers with a passing score  (> 9 of 18 correct answers) | Odds ratio: 10.9  (CI 95% 4.4-27.2)  Based on data from 212 teachers in 3 studies | **615**  per 1000 | **945**  per 1000 | **Moderate**  Due to missing outcome data^§^ and insufficient sample size** | The IHC secondary school intervention may increase the proportion of teachers with a passing score after one year. |
|  |  | Difference: **330 more per 1000**  (CI 95% 260-362 more) | |  |  |
| Teachers with a mastery score  (> 14 of 18 correct answers) | Odds ratio: 24.7  (CI 95% 11.0-55.2)  Based on data from 212 teachers in 3 studies | **125**  per 1000 | **779**  per 1000 | **Moderate**  Due to missing outcome data^§^ and insufficient sample size** | The IHC secondary school intervention may increase the proportion of teachers with a mastery score after one year. |
|  |  | Difference: **654 more per 1000**  (CI 95% 486-762 more) | |  |  |
| Teachers' mean score | Based on data from 212 teachers in 3 studies | **54.0**  Mean | **81.0**  Mean | **Moderate**  Due to missing outcome data^§^ and insufficient sample size** | IHC secondary school intervention may improve teachers' mean score after one year. |
|  |  | Difference: **MD 27.0 higher**  (CI 95% 22.6-31.3 higher) | |  |  |

|  |  | Difference: **MD 27.0 higher**  (CI 95% 22.6-31.3 higher) |  |  |
| --- | --- | --- | --- | --- |

* Clustering was accounted for using random intercepts at the level of trial and, for outcomes measured on students. We used generalized linear mixed models to perform individual participant data (IPD) meta-analyses, using the original data and inverse probability weights from the three trials. We estimated adjusted odds ratios for students and teachers achieving passing and mastery scores (logistic regression: logit link, Bernoulli errors) and adjusted differences in students' and teachers' mean scores (linear regression: identity link, normal errors), assuming common treatment effects across the trials. We adjusted for school ownership (public or government-funded versus private), use of a projector (versus blackboard) and school performance as fixed effects to account for the stratification used in the original trials as planned. The absolute effect estimates are based on the control school (standard national curriculum) results and the control school results plus the adjusted difference for the intervention schools.

† We rated the certainty of evidence for the primary outcome in relation to a threshold of at least 20% more students with a passing score. For secondary outcomes, we rated the certainty of evidence in relation to null effect. The initial assessments (for results just after the intervention) were made by two independent judges (Xiajing Chu and Liam Yao).

‡ The effective sample size accounting for cluster is the original sample size divided by a quantity called the ‘design effect’.^1^

§ We modified the assessments of risk of bias to serious due to missing outcome data (see Table S14). The point estimates appear unbiased based on sensitivity analyses. However, 27% of the students and 13% of the teachers enrolled in the trials did not complete the CTH test at one year, and we cannot rule out that this biased the results to some degree. In addition, the confidence intervals were wider due to missing outcome data and the 95% confidence intervals for the primary outcome (passing scores) crossed the 20% threshold (the pre-specified smallest important difference) in the random effects and inverse probability weighted meta-analyses.

** Serious imprecision due to insufficient sample size. Although we cannot rule out some degree of bias due missing outcome data for 13% of the teachers enrolled in the trial, the point estimates appear to be robust based on sensitivity analyses and the lower confidence limits all suggest a large effect, so we did not downgrade the certainty of the evidence twice (to low) due to insufficient sample size and missing data, both of which may have resulted in imprecision.

**Reference**

Higgins JPT, Eldridge S, Li T (editors). Section 23.1.4 Approximate analyses of cluster-randomized trials for a meta-analysis: effective sample sizes. In: Higgins JPT, Thomas J, Chandler J, et al. (editors). *Cochrane Handbook for Systematic Reviews of Interventions*version 6.3 (updated February 2022). Cochrane, 2022. Available from [www.training.cochrane.org/handbook](http://www.training.cochrane.org/handbook).

# Figure S1. Students with a mastery score (≥14 out of 18) at one-year follow-up


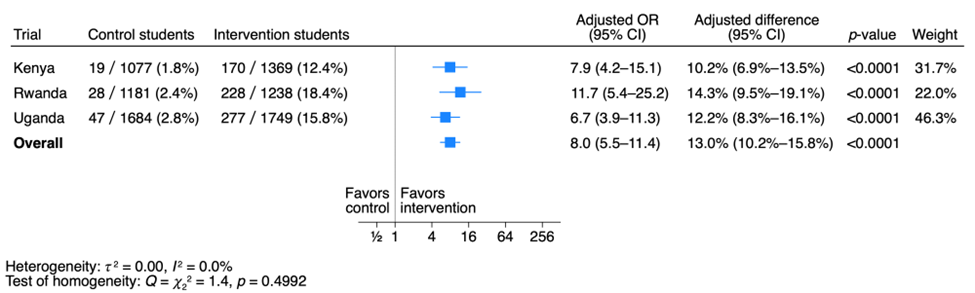


# Figure S2. Students’ mean score at one-year follow-up


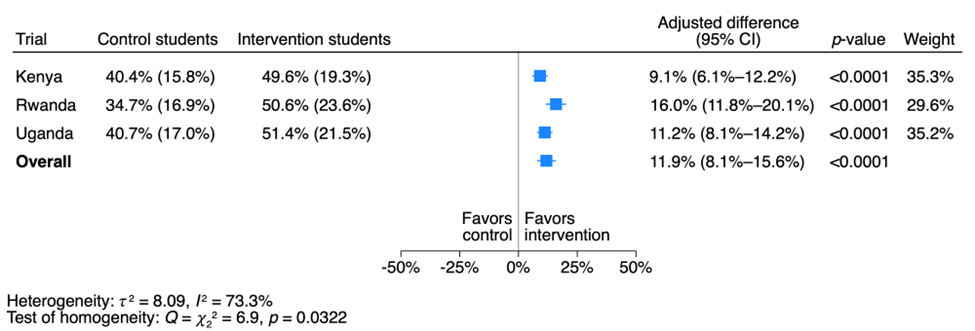


# Figure S3. Teachers with a passing score


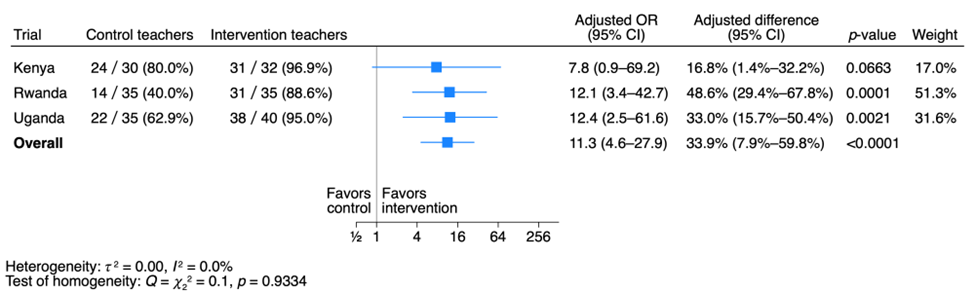


The standard for a passing score was ≥ 9 of 18 correct answers. The overall adjusted odds ratio was calculated using inverse variance-weighted random effects meta-analysis. The adjusted differences were calculated using the control odds and the adjusted odds ratios, accounting for uncertainty of the control odds as well as the odds ratios.

# Figure S4. Teachers with a mastery score


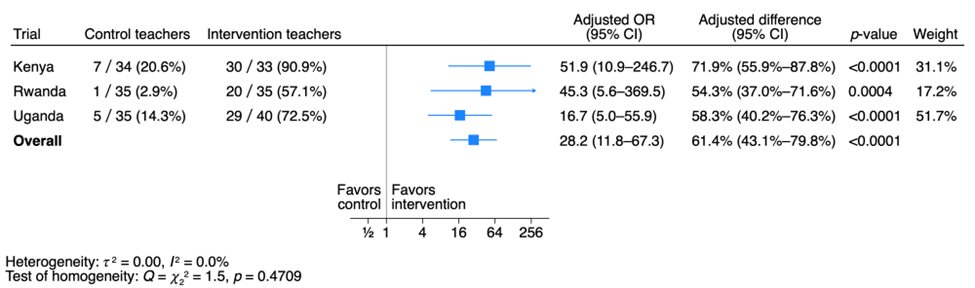


The standard for a score indicating mastery was ≥ 14 of 18 correct answers. The overall adjusted odds ratio was calculated using inverse variance-weighted random effects meta-analysis. The adjusted differences were calculated using the control odds and the adjusted odds ratios, accounting for uncertainty of the control odds as well as the odds ratios.

# Figure S5. Teachers' mean score


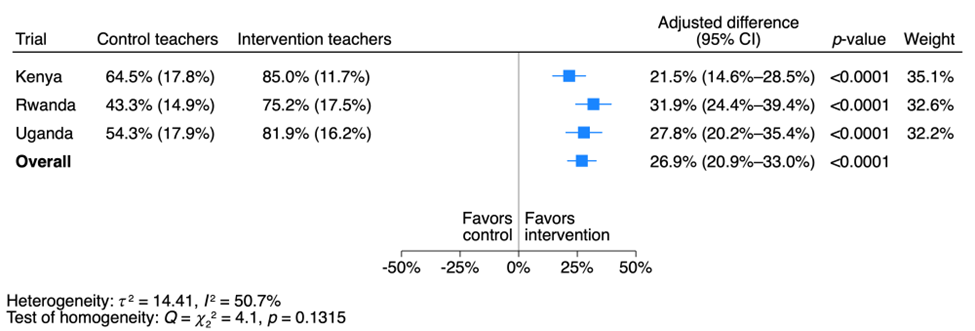


# Appendix 1. GREET checklist

Guideline for reporting evidence-based practice educational interventions and teaching (GREET) checklist^1^

BRIEF NAME

1. **Intervention:** Informed Health Choices (IHC) secondary school intervention

The intervention (providing teacher training and the [*Be Smart about your Health*](https://besmarthealth.org/) digital resources) was compared to routine practice (teaching according to the national lower-secondary school curriculum without intervening).

WHY this educational process

2. **Theory:** The IHC secondary school intervention is based on the *IHC Key Concepts* framework. The framework includes concepts (principles) that people should understand and apply when deciding whether to believe a claim about the effects of health actions (things that people do to care for their health or the health of others) and what to do.^2,3^ The framework is based on evidence of the importance of the included concepts,^4,5^ logic, feedback, other relevant frameworks,^6^ and adaptation of the IHC Key Concepts to other types of interventions such as educational, environmental, and policing interventions.^7^

We developed the resources between 2020 and 2022 using a human-centered design approach.^8^ This included cycles of idea generation and prototyping, piloting with observation, user-testing with teachers and students, and feedback from teachers, students, and curriculum developers in Kenya, Rwanda, and Uganda, and an international advisory group. The aim of the design process was to ensure that teachers and students find the intervention to be engaging and useful, and the resources easy to use.

The teaching strategies used in the resources were based in part on an overview of systematic reviews of teaching strategies,^9^ and draw on several educational theories. These include social constructivist theory (which postulates that learning can be maximized through well-designed, intentional social interaction with other learners),^10^ the theory of active student response (which postulates that learning is enhanced by high levels of active student response),^11^ and the elaborative retrieval hypothesis (which postulates that the search for correct answers on practice tests or quizzes results in multiple retrieval routes which aid later recall).^12^

3. **Learning objectives:** The primary learning goal is for students to have a basic ability to think critically about health actions and understand why this is important. They should be able to recognize claims about the effects of health actions and assess some of those claims. They should understand why it is important for them that researchers study the effects of health actions and recognize two key features of reliable comparisons of health actions. They should recognize that health actions can have both advantages and disadvantages and the importance of weighing the benefits and savings against the harms and costs when deciding what to do.

4. **Evidence-based practice content:** The resources focus on nine IHC Key Concepts that were prioritized by curriculum developers, teachers, and researchers in Kenya, Rwanda, and Uganda.^13^

WHAT

5. **Materials:** The resources are open access digital resources for lower-secondary school teachers. They include 10 lesson plans. There are two versions of each lesson plan: one for teachers who are using a blackboard or similar (e.g., whiteboard or flipchart), and one for teachers using a projector, in the classroom. The aim is for students to learn to think critically about health claims and choices. We provided teachers in the intervention schools with a link to the resources, which they could access via a web browser, and download for offline use, in the same web browser. The resources are responsive, which means the design adapts to the device, including computers and smartphones with different screen sizes. Schools in both the control and intervention group continued teaching the national curricula, which did not explicitly include teaching critical thinking about health. We did not provide any additional materials to the control schools.

Each lesson plan includes an introduction, an activity, and a wrap-up. The introduction includes the key messages from the previous lesson, a discussion question about the previous lesson, and what the new lesson is about. The activity is designed to help students achieve the learning goals. The wrap-up includes a question about what the students learned, the key messages for the lesson, sometimes a homework assignment, and what the next lesson is about. Lessons 5 and 10 include quizzes and discussions of application of what students learned in their daily lives.

Each lesson plan is accompanied by an overview and background for teachers. The overview includes learning goals, key terms introduced in the lesson, and the main teaching strategies used in the lesson. The background includes a description of what the lesson is about and if relevant, explanations of common misunderstandings and closely related content that is not covered in the lesson.

In addition, there is a teachers’ guide, materials for teacher training workshops, information about how to use the resources (help), optional printouts (PDFs) for teachers and students, and a glossary. We provided teachers with binders with printouts at the training workshops.

6. **Educational strategies:** Key strategies used across lessons included guided note taking, small group discussion, use of response cards,^11^ homework, use of a standard lesson structure, setting objectives and providing feedback, and – for lessons using a projector - multimedia design. Other strategies used in some of the lessons include concept cartoons, inquiry-based instruction, and role play.

7. **Incentives:** The incentive for teachers and students was the value they perceived in learning to think critically about health actions. We reimbursed teachers at schools without Internet access for the cost of downloading the resources and any other costs related to participation in the trial. They were not paid for participating in the trial and there were no other financial incentives for the schools, head teachers, teachers, or students. The evaluation administered at the end of the school term did not count towards the students’ school marks or assessment of the teachers or schools.

WHO PROVIDED

8. **Instructors**: The head teacher at each participating school selected a teacher of a relevant subject (e.g., biology) for year-1 or year-2 of lower-secondary school. The teachers were invited to a 2-3-day workshop to introduce them to the resources and the learning content. The training was facilitated by other teachers who had participated in one of the teacher networks that helped to develop the resources or who piloted use of the resources. The facilitators were provided with presentations and other materials for the workshops, and they reviewed the material and plans for the workshops with the research teams prior to the workshops.

HOW

9. **Delivery:** Schools decided whether teachers would deliver the 10 lessons during or outside of regular classroom time. Teachers could use a computer, smartphone, or printouts when delivering the lessons. Depending on what equipment was available to the teachers, they delivered the lessons to students using only a blackboard or using a projector and slide presentations that are included in the digital resources. The number of students in a class varied.

WHERE

10. **Environment:** We recruited representative samples of schools, including rural and urban schools. The conditions in the schools varied. Details of the contexts in each of the three countries can be found in reports of the context analyses undertaken prior to developing the resources.^14-16^

WHEN and HOW MUCH

11. **Schedule:** The 10 lessons were taught in a single school term. Each school decided how to fit the lessons into the schedule for that term.

12. **Amount of time**: Each lesson is designed to be delivered in a single period (40 minutes) but may take more time. Teachers were instructed to encourage students to collect and assess claims about the effects of health actions outside of class and to discuss claims with their families and friends. The teachers needed up to 30 minutes to prepare for each lesson.

PLANNED CHANGES

13. **Adaptation**: No specific adaptation was required, but teachers were able to adapt the lessons, for example by using different or additional examples or editing the presentations.

UNPLANNED CHANGES

14. **Modifications**: As part of the process evaluations, we asked teachers to complete an evaluation form after each lesson, including information about changes they made to the lesson plan, and we observed each teacher for one lesson. We did not give feedback to the teachers during the trial.

HOW WELL

15. **Attendance:** The teachers were asked to record attendance for each lesson and to encourage students to attend all lessons by telling them when the next lesson would be and its learning goals. We designed the lessons to appeal to students and to make clear the relevance and importance of the learning goals.

16. **Fidelity**: We will explore the extent to which teachers delivered the lessons as planned in the process evaluation, based on the evaluation forms completed by teachers after each lesson, observations of their teaching a lesson, and interviews with teachers and students.

17. **Delivery schedule**: We asked the teachers to record when each lesson was taught, the duration of each lesson, and whether all the lessons were completed as planned.

**References**

1. Phillips AC, Lewis LK, McEvoy MP, et al. Development and validation of the guideline for reporting evidence-based practice educational interventions and teaching (GREET). *BMC Med Educ* 2016; 16: 237.

2. Oxman AD, Chalmers I, Austvoll-Dahlgren A, Informed Health Choices Group. Key Concepts for assessing claims about treatment effects and making well-informed treatment choices. *F1000Res* 2019; 7: 1784.

3. Oxman AD, Chalmers I, Dahlgren A, Informed Health Choices Group. Key Concepts for assessing claims about treatment effects and making well-informed treatment choices. Version 2019. *IHC Working Paper* 2019.

4. Oxman AD, Chalmers I, Dahlgren A, Informed Health Choices Group. Key Concepts for Informed Health Choices: a framework for enabling people to think critically about health claims (Version 2022). *IHC Working Paper* 2022.

5. Oxman AD, Chalmers I, Dahlgren A. Key Concepts for Informed Health Choices: Where’s the evidence? *F1000Res* 2022; 11: 890.

6. Oxman AD, Martinez Garcia L. Comparison of the Informed Health Choices Key Concepts to other frameworks that are relevant to learning how to think critically about treatment claims, comparisons, and choices: protocol for a mapping review. *IHC Working Paper* 2018.

7. Aronson JK, Barends E, Boruch R, et al. Key concepts for making informed choices. *Nature* 2019; 572(7769): 303-6.

8. Rosenbaum SE, Moberg J, Chesire F, et al. Teaching critical thinking about health information and choices in secondary schools: human-centred design of digital resources. *F1000Res* 2023.

9. Oxman AD, Nsangi A, Martinez Garcia L, et al. The effects of teaching strategies on learning to think critically in primary and secondary schools: an overview of systematic reviews. *Review of Education* Submitted 20 January 2023.

10. Igel C. Cooperative learning. Denver, CO: McREL International, 2010.

11. Randolph JJ. Meta-analysis of the research on response cards: effects on test achievement, quiz achievement, participation, and off-task behavior. *J Posit Behav Interv* 2007; 9(2): 113-28.

12. Pan SC, Rickard TC. Transfer of test-enhanced learning: meta-analytic review and synthesis. *Psychol Bull* 2018; 144(7): 710-56.

13. Agaba JJ, Chesire F, Mugisha M, et al. Prioritisation of Informed Health Choices (IHC) Key Concepts to be included in lower-secondary school resources: a consensus study. *PLoS One* 2023: in press.

14. Mugisha M, Uwitonze AM, Chesire F, et al. Teaching critical thinking about health using digital technology in lower secondary schools in Rwanda: A qualitative context analysis. *PLoS One* 2021; 16(3): e0248773.

15. Ssenyonga R, Sewankambo NK, Mugagga SK, et al. Learning to think critically about health using digital technology in Ugandan lower secondary schools: a contextual analysis. *PLoS One* 2022; 17(2): e0260367.

16. Chesire F, Ochieng M, Mugisha M, et al. Contextualizing critical thinking about health using digital technology in secondary schools in Kenya: a qualitative analysis. *Pilot and Feasibility Studies* 2022: Forthcoming.

# Appendix 2. The Critical Thinking about Health test

**Instructions**

*Before you start, please note that some words in this questionnaire may not be familiar to you. Please read through the following explanations:*

A **TREATMENT** is anything done to care for yourself, so you stay well or, if you are sick or injured, so you get better and not worse. For example, skin cream.

A **TREATMENT CLAIM** is something someone says about whether a treatment causes something to happen or to change. A claim can be true or can be false. For example, if a friend says “Using skin cream will help your skin rash”.

A **RESEARCH STUDY** is a way to answer a question by carefully collecting information. For example, a study might be done to answer the question: Does skin cream help people with skin rash?

**RESULTS of a study** are what the study found. For example, whether people who use skin cream had less skin rash.

When something happens by **CHANCE**, it is not possible to tell in advance what will happen. For example, if you flip a coin, you cannot tell in advance if it will land on one side or the other side.

First, read the text above the questions and then answer each question on **the SCORE sheet,** using one of the provided answers.

For each question, choose what you think is the best answer and

**fill in the circle** for that answer in the score sheet, like this.


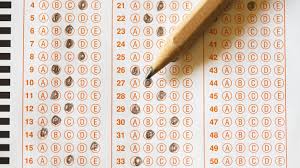


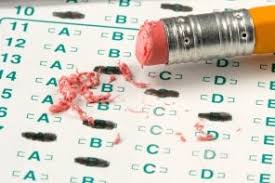


**If you want to change your answer,** carefully erase the first circle that you filled in.


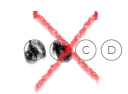
Do not fill in more than one circle for each question.

The examples below show you the one correct way and some wrong ways to mark your answers.

**Be sure to fill in the circles the correct way.**


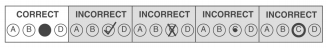


**Part 1.**

**Questions about you**

**1.1 Your school code**

**1.2 Your teacher code**

**1.3 Your gender** ☐ Female

☐ Male

**1.4 Your age**

| A doctor did a research study to find out if drinking tea keeps people from getting sick. He flipped a coin to decide who should get the tea and who should not. People who got tea went to the doctor’s office every day to drink their tea. At the end of the study, people who got the tea were less likely to be sick than those who got no tea.  *Based on the text above, please answer the following questions:* |
| --- |
| **2.1 Who went to the doctor’s office every day?** |
| *Options:*   1. People who did not get tea 2. People who got tea 3. Everyone 4. People who got sick |
|  |
| **2.2 How did the doctor decide who should get tea?** |
| *Options:*   1. By flipping a coin 2. By asking people if they would like tea 3. The doctor gave tea to those who were more likely to be sick 4. The doctor asked people who came to his office |
|  |

**Part 2. Reading ability questions**

| A doctor did a research study to find out if drinking tea keeps people from getting sick. He flipped a coin to decide who should get the tea and who should not. People who got tea went to the doctor’s office every day to drink their tea. At the end of the study, people who got the tea were less likely to be sick than those who got no tea.  *Based on the text above, please answer the following questions:* |
| --- |
| **2.3** **What was the treatment?** |
| *Options:*   1. Tea 2. Sleep 3. The study 4. The doctor |
|  |
| **2.4 What was the result of the study?**  *Options:*   1. Drinking tea can help people from getting sick 2. Doctors should toss coins when doing studies 3. People should go to the doctor if they are sick 4. Not drinking tea can help people from getting sick |
|  |

**Part 3.**

**Questions about claims**

| **3.1** |
| --- |
| Anne has pain in her ear, and she asks her brother. Hassan what to do about it. He says that once, when he had a pain like that, he cleaned his ear with hot water. The next day, his ear pain was gone. Based on his experience, he says rinsing with hot water is helpful for ear pain. |
| *Question:* **Do you agree with Hassan?** |
| *Options:*   1. Yes. Because this is Hassan’s experience, it is likely to be true 2. No, Hassan’s experience is not enough to be sure 3. Yes, Hassan rinsed his ear with hot water and the next day his ear pain was gone |

| **3.2** |
| --- |
| Sarah says that medicines from well-known companies, costing more money, are not necessarily the best. Medicines from less known companies, costing less money, may be just as good or even better. |
| *Question:* **Is Sarah right?** |
| *Options:*   1. No, medicines costing less money are more likely to be harmful than expensive medicines 2. Yes, just because the medicine is expensive does not mean that it will work better than other medicines 3. No, expensive medicines made by well-known companies are better than less expensive medicines made by lesser-known companies |

| **3.3** |
| --- |
| Edith has stomach pain. Edith’s mother says that fruit juice is a good treatment for stomach pain. She learnt about this treatment from Edith’s grandmother. Over many years, other families she knows have also used fruit juice to treat stomach pain. |
| *Question:* **Based on this, how sure can we be that fruit juice is a good treatment for stomach pain?** |
| *Options:*   1. Not very sure. Even though people have used fruit juice over many years, that does not mean that it helps stomach pain 2. Very sure. If it has worked for Edith’s mother and other people who have tried it, it will probably work for her too 3. Not very sure. Edith should ask more families if they use fruit juice to treat stomach pain |

| **3.4** |
| --- |
| John has a skin rash on his leg. A shop sells several skin creams to treat skin rashes. John chooses a skin cream from a well-known company, even though it is more expensive than the other creams. John thinks this skin cream is more likely to heal his rash than the other skin creams because it is more expensive. |
| *Question:* **Is John right?** |
| *Options:*   1. No, just because the skin cream is expensive does not mean that it will work better than other creams 2. It is not possible to say. However, expensive skin creams are likely to be better because the companies spend more time making them 3. No, the skin cream is probably not as good as the other skin creams. People just like well-known companies more |

| **3.5** |
| --- |
| Sarah has a sickness. There is a medicine for it, but she is not sure if she should try it. A research study comparing the medicine with no medicine found that the medicine was helpful but also that it could be harmful. Three of Sarah’s friends are telling her what to do. |
| *Question:* **Which of the following things said by her friends is more correct?** |
| *Options:*   1. She should only take the medicine if many people have tried the medicine before 2. She should only take the medicine if she thinks it will help her more than it will harm her 3. If Sarah has enough money to buy the medicine, it could not hurt to try it |

| **3.6** |
| --- |
| Imagine you and your friends have formed a team to take part in a local running competition. People on the other teams all had bananas for breakfast. You and your friends did not have bananas for breakfast and lost the race. Some people say that this was because your team had bread for breakfast and that made them run slower. |
| *Question:* **If you did a research study comparing people who eat bananas for breakfast with people who don’t eat bananas for breakfast, how would you decide who should have bananas for breakfast?** |
| *Options:*   1. By chance (like flipping a coin) to make sure the two groups are as similar as possible 2. By having the teams decide, to make it as fair as possible 3. By having the teachers decide, because they know who would benefit best from eating bananas |

| **3.7** |
| --- |
| Regina has a sickness that makes it difficult for her to breathe. She hears on the radio about a medicine that has helped many people with breathing problems. |
| *Question:* **How sure can Regina be that the medicine does not have any harms?** |
| *Options:*   1. It is not possible to say, it depends on how much hope Regina has in the medicine 2. Very sure, since the medicine has helped many people, it is unlikely that it also harms people 3. Not very sure, because all medicines may harm people as well as help them |

| **3.8** |
| --- |
| Outside the city where Paul lives there are many farms. The farmers often get coughs. For many years, the farmers have used strong tea to treat their coughs. They say that the tea is good for them and that it protects them from becoming more sick.  Paul says that the farmers may not be right, and that the strong tea may not help coughs. |
| *Question:* **Do you agree with Paul?** |
| *Options:*   1. Yes, Paul should try drinking strong tea himself to know for sure. The strong tea may work differently on him 2. Yes, we can only know for sure if the strong tea works if it has been compared with other treatments in studies 3. No, the farmers would not have used strong tea for all those years if it did not work |

| **3.9** |
| --- |
| Jane often has headaches. Her doctor tells her that there is a medicine that may help her, but it may harm her. The medicine is also very expensive. |
| *Question:* **What does Jane need to think about before using the medicine?** |
| *Options:*   1. If the medicine will help her more than it will hurt her, and if she thinks it is worth paying so much money for it 2. If anybody she knows has tried the medicine so that she can ask them what they thought about it 3. If she should ask another doctor, since the doctor must be wrong. A medicine which is helpful cannot be harmful |

| **3.10** |
| --- |
| Mercy wanted to know if eating bananas makes you run faster. To find out, she invited her six best friends to take part in a research study. Three friends each got bananas, and three friends did not get bananas. At the end of the study, the friends who did not get bananas ran a lot faster. |
| *Question:* **How sure can Mercy be about her study’s results?** |
| *Options:*   1. More sure, because Mercy found a difference between the groups in how fast they ran. This means that the study included enough people. 2. Less sure, because the difference between the two groups could have occurred by chance 3. More sure, if she repeats the study with six more friends |

| **3.11** |
| --- |
| Doctors studied people with stomach pain before and after they took a new medicine. After taking the new medicine, many people felt less pain. |
| *Question:* **Can we be sure that the new medicine is good for treating stomach pain?** |
| *Options:*   1. No, taking the new medicine should have been compared either with not taking the medicine, or with taking an older medicine 2. Yes, people were asked how much pain they felt before and after they took the new medicine 3. Yes, the study was done by doctors |

| **3.12** |
| --- |
| A new and an old mosquito spray (insecticide) were compared in a research study. In the study, two houses were sprayed with the new spray, and two houses were sprayed with the old spray. Based on this study, the new spray was better for protecting against mosquito bites than the old spray. Neither of the sprays was found to be harmful to people. |
| *Question:* **How sure can you be about what the study found?** |
| *Options:*   1. Less sure, because only four houses were studied and the differences between sprays may have happened by chance 2. More sure, because the new spray was better for protecting against mosquito bites and it was not harmful 3. More sure, because the new spray was found to be better, and the differences between sprays is unlikely to have happened by chance |

| **3.13** |
| --- |
| On the radio, there is someone selling a treatment - a new juice. The seller says that if you drink one glass of it every day, you will not get sick. |
| *Question:* **How sure can you be that the new juice will keep you from getting sick?** |
| *Options:*   1. It is not possible to say. I would have to try the new juice myself to be sure 2. Very sure, otherwise this news would not be on the radio 3. Not very sure. Very few treatments work so well |

| **3.14** |
| --- |
| Dr. Javier has done a research study giving a new medicine to people who were vomiting. Some of the people stopped vomiting after they got the new medicine. Dr. Javier says that this means that the medicine works. |
| *Question:* **Is Dr. Javier right?** |
| *Options:*   1. No. The people who used the medicine were not compared with similar people who did not use the medicine 2. Yes, some of the people stopped vomiting 3. No, since not all the people stopped vomiting |

| **3.15** |
| --- |
| George has stomach pain. The last time George had a stomach pain was two months ago. That time, he drank some hot milk and after an hour, his stomach pain was gone. Therefore, George says hot milk cures stomach pain. |
| *Question:* **Is George right?** |
| *Options:*   1. It is not possible to say. His stomach pain might have gone away without the hot milk 2. It is not possible to say, but it is likely to be true based on the fact that George had this experience 3. Yes, George’s experience is enough to show that hot milk makes stomach pain go away |

| **3.16** |
| --- |
| Esther recommends a new treatment – a medicine - for pain. She says that everyone who has tried it felt better. |
| *Question:* **How sure can you be that what Esther says about the new medicine is true?** |
| *Options:*   1. Not very sure. Very large benefits, where everyone or nearly everyone gets better because of a treatment are rare 2. It is not possible to say. To be sure I would have to try the medicine for myself 3. Very sure. The medicine must be very good since everyone who has tried it got better |

| **3.17** |
| --- |
| A doctor wanted to know which of two treatments was best for headaches. In a study to find out, he asked people to choose which treatment they would like to get. He compared the people who took each of the two treatments. |
| *Question:* **How sure can we be about the results of this comparison of the two treatments?** |
| *Options:*   1. More sure, because the doctor asked people to choose which treatment they wanted 2. Less sure, because the doctor should have decided who got which treatment 3. Less sure, because the doctor should have given people one of the two treatments by chance (like flipping a coin) |

| **3.18** |
| --- |
| Mary wanted to find out which plants were best for treating people with headaches, so she did a research study to compare green plants with yellow plants. The people who used the green plants had fewer headaches compared to the people who used the yellow plants. |
| *Question:* **How sure can we be that green plants are better than yellow plants?** |
| *Options:*   1. It is not possible to say. Mary did not study possible harms of the plants 2. Very sure, since people who used the green plants had fewer headaches 3. Not very sure, it depends on how much people believe the green plants will work |

**Part 4. Questions about your views**

Below are some questions about what you think. **There are not right or wrong answers to these questions.**

Below are some actions. Please read each one carefully and give the answer that comes closest to how difficult or easy you find each of the actions to be. There are not right or wrong answers to these questions.

4.1 *Question:* How difficult or easy do you find knowing if a claim about a treatment is based on a research study comparing treatments?

*Options:*

1. Very difficult
2. Difficult
3. Easy
4. Very easy
5. I don’t know

4.2 *Question:* How difficult or easy do you think it is to find information about treatments that is based on research studies comparing treatments?

*Options:*

1. Very difficult
2. Difficult
3. Easy
4. Very easy
5. I don’t know

Below are some actions. Please read each one carefully and give the answer that comes closest to how difficult or easy you find each of the actions to be. There are not right or wrong answers to these questions.

4.3 *Question:* How difficult or easy do you find judging the trustworthiness of the results of a research study comparing treatments?

*Options:*

1. Very difficult
2. Difficult
3. Easy
4. Very easy
5. I don’t know

4.4 *Question:* How difficult or easy do you find knowing if the results of a research study comparing treatments are relevant to you?

*Options:*

1. Very difficult
2. Difficult
3. Easy
4. Very easy
5. I don’t know

Think about a sickness that you might get. Imagine someone claiming (saying) that a treatment might help you get better.

4.5 *Question:* How likely are you to find out what the claim was based on (for example by asking the person making the claim)?

*Options:*

1. Very unlikely
2. Unlikely
3. Likely
4. Very likely
5. I don’t know

4.6 *Question:* How likely are you to find out if the claim was based on a research study comparing the treatment to no treatment?

*Options:*

1. Very unlikely
2. Unlikely
3. Likely
4. Very likely
5. I don’t know

4.7. *Question:* How likely are you to say “yes” if you are asked to participate in a research study comparing two treatments for your sickness?

*Options:*

1. Very unlikely
2. Unlikely
3. Likely
4. Very likely
5. I don’t know

**Part 5.**

**Questions about your experience with the Be Smart about Your Health lessons**

Below are some questions about what you think. **There are not right or wrong answers to these questions.**

| **5.1.** *Question:* **How much did you like or dislike the lessons?** |
| --- |
| *Options:*   1. I liked the lessons very much 2. I liked the lessons a little 3. I disliked the lessons a little 4. I disliked the lessons very much |
| **5.2.** *Question:* **How easy or difficult were these lessons to understand?** |
| *Options:*   1. Very difficult to understand 2. Difficult to understand 3. Easy to understand 4. Very easy to understand |
| **5.3.** *Question:* **How helpful or unhelpful has what you have learned been to you?** |
| *Options:*   1. Very helpful to me 2. Helpful to me 3. Unhelpful to me 4. Very unhelpful to me |

# Appendix 3. Additional questions about transfer and adverse effects

As noted in the protocol, we planned to assess potential adverse effects and use of what was learned in daily life (far transfer) in the one-year follow-up studies. Based on preliminary findings from interviews with teachers and students,^1^ and user testing potential questions, we identified the following potential adverse effects that we assessed quantitatively: conflict due to students who participated in the lessons challenging the beliefs of others, stress, decision-making harms due to misunderstandings, and waste. Questions to assess these outcomes were included in questionnaires for students and teachers. These questionnaires were completed by participants in the intervention schools only. The questions that address each of the potential adverse effects are shown below.

***Questions about students and teachers experience with the “Be Smart about Your Health” lessons***

|  | **Questions** | |
| --- | --- | --- |
|  | **Students** | **Teachers** |
| Waste | 1-3 | 5-8 |
| Conflict due to students challenging the beliefs of others | 4-6 | 3 |
| Stress | 7 | 1-2 |
| Decision-making harms due to misunderstandings |  | 4 |
| Other | 8 |  |

**Reference**

1. Oxman M, Chesire F, Mugisha M, Ssenyonga R, Ngatia B, Nsangi A, et al. Potential adverse effects of an educational intervention: Development of a framework. Glob Health Sci Pract. 2024; submitted.


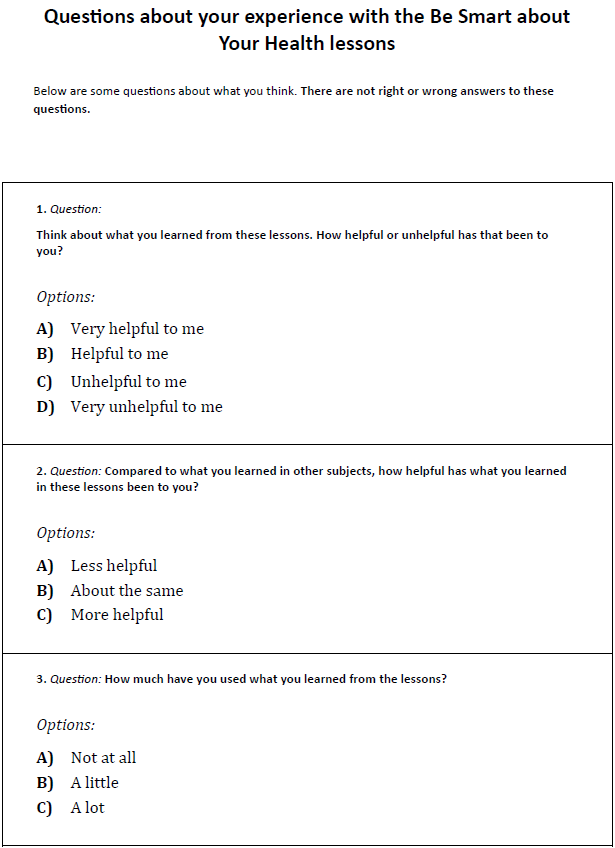
Additional questions for students


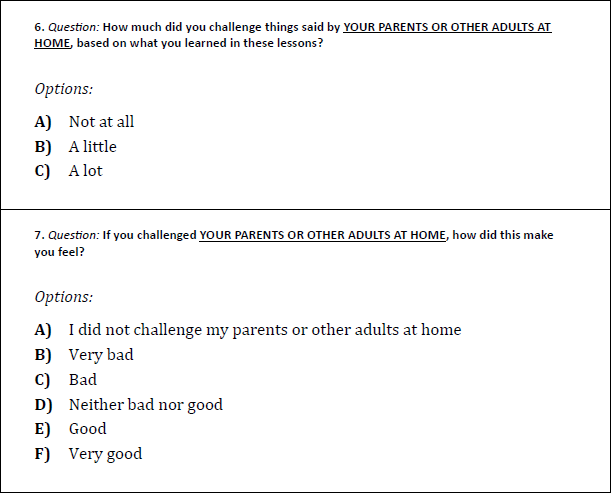

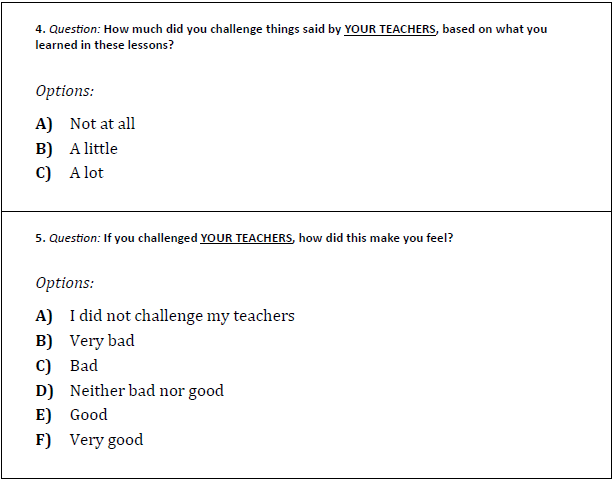


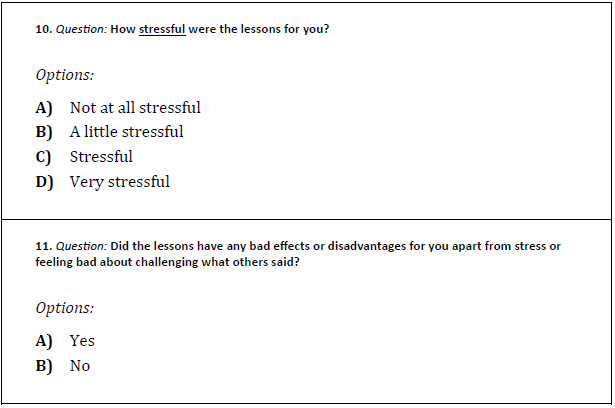

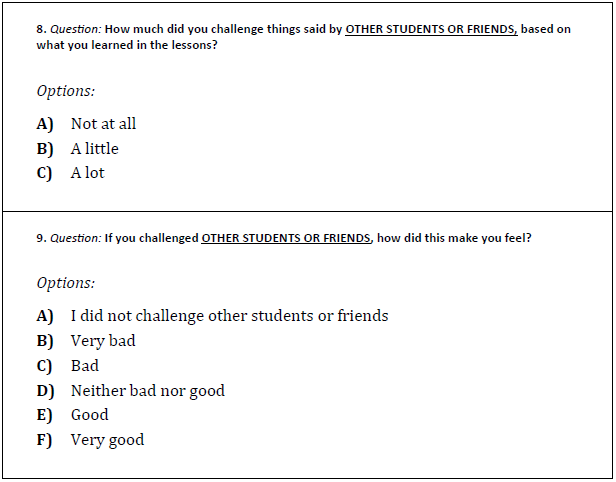


Additional questions for teachers


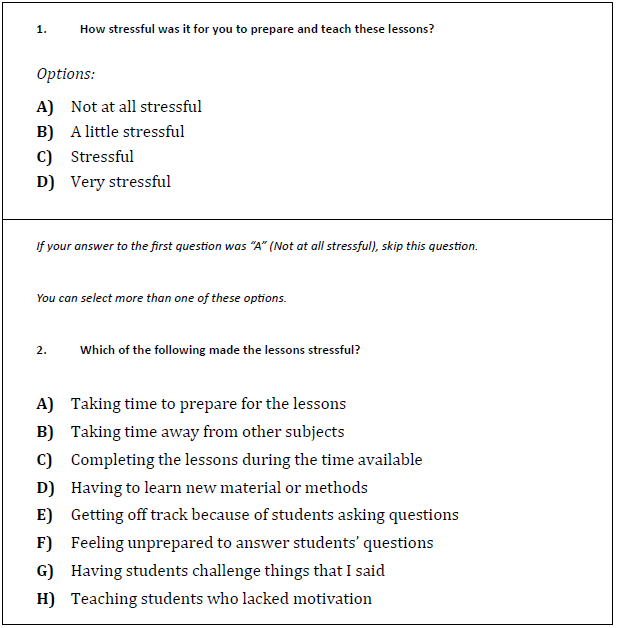


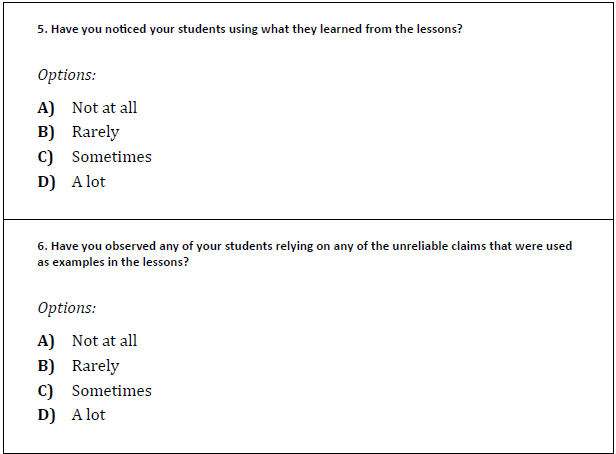

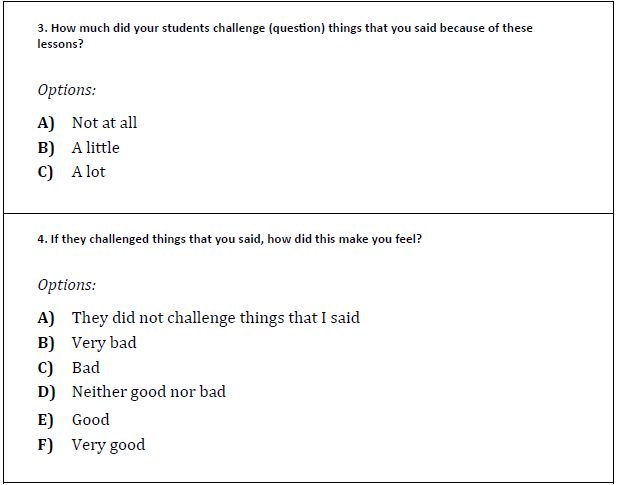


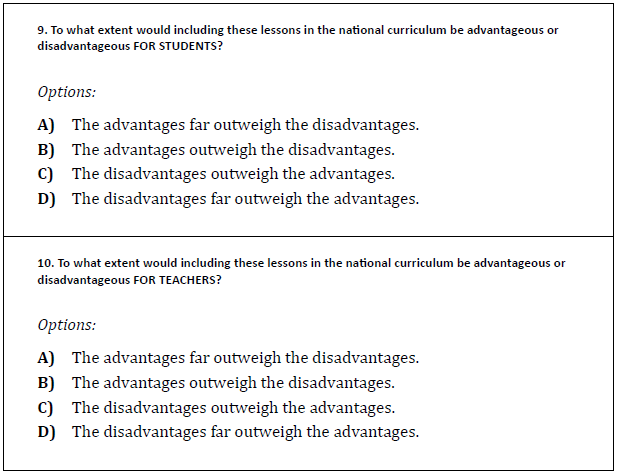

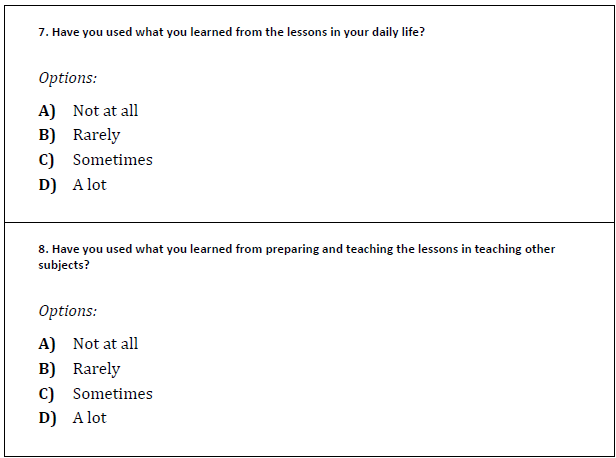


# Appendix 4. Critical thinking about health diary

Critical Thinking about Health

**Definitions**

| *Some words in this diary may not be familiar to you or may be used in a different way than you are used to.*  **HEALTH** is how well someone’s body and mind are.  A **HEALTH ACTION** is something that someone does to care for their health or the health of others.  An **EFFECT OF A HEALTH ACTION** is a change in health caused by the action.  A **CLAIM** is something that someone says as if it is true, but it may be wrong.  A **RELIABLE CLAIM** is a claim that can be trusted or relied on.  An **UNRELIABLE CLAIM** is a claim that cannot be trusted or relied on.  A **CLAIM ABOUT THE EFFECTS OF A HEALTH ACTION** is something that someone says as if it is true - but it may be wrong - about what will happen if you take a certain health action.  The **BASIS FOR A CLAIM** is the information used to support the claim. |
| --- |

**What is TODAY’S DATE? ________________________**

**Questions about you**

*Please answer the following questions:*

**1. What is your school’s code? ________________________**

**2. What is your gender?** ☐ Female

☐ Male

**3. What is your age? ________________________**

**Questions about the past week**

*Think about a* **CLAIM ABOUT THE EFFECTS OF A HEALTH ACTION** *that you heard, read, made, or thought about during the past week.* ***Then answer the questions on the next page.***

Here are some examples of claims about the effects of a health action:

- *A friend told me “After I put toothpaste on my pimples, the pimples went away. So, toothpaste removes pimples.”*
- *“Putting cow dung on a burn will heal the burn.” People have been doing that for a long time, so it must work.*
- *I heard “There is a new, expensive flu medicine that cures the flu.”*

*Write* ***one*** *CLAIM* ***that you, heard, read made, or thought about during the past week*** *here.* ***DO NOT USE ONE OF THE CLAIMS ABOVE****.*

____________________________________________________________________________________

____________________________________________________________________________________

____________________________________________________________________________________

____________________________________________________________________________________

____________________________________________________________________________________

____________________________________________________________________________________

____________________________________________________________________________________

____________________________________________________________________________________

***For the CLAIM that you heard, read, made, or thought about:***

**1. What was the HEALTH ACTION in that claim? ____________________________**

____________________________________________________________________________________

**2. What was the CLAIMED EFFECT in that claim? ___________________________**

____________________________________________________________________________________

**3. Was there a BASIS FOR THAT CLAIM? 🞎 Yes**

**🞎 No**

**If yes, what was the basis for that claim? __________________________**

____________________________________________________________________________________

____________________________________________________________________________________

**4. How reliable do you think that claim is?** **🞎 Reliable**

**🞎 Can’t tell**

**🞎 Unreliable**

**5. Would you take that health action, or would you advise someone else to take that health action?**

**🞎 Yes**

**🞎 No**

**6. If you answered YES, why would you?**

**If you answered NO, why wouldn’t you?**

**____________________________________________________________________________________**

____________________________________________________________________________________

____________________________________________________________________________________

____________________________________________________________________________________

# Appendix 5. Rubric for scoring the diary

We coded the students’ responses to the questions in the *Critical thinking about health diary* as follows:

1. Was the action correctly identified?

1. Yes
2. No

2. Was the claimed effect correctly identified?

1. Yes
2. No

If either the action (1) or the claimed effect (2) was not correctly identified, stop here. In the analysis, we will consider the assessment of the reliability of the claim (question 4 below) to be incorrect for these students and we will exclude them from the other analyses.

3. Was the basis for the claim correctly identified?

1. There was not a basis for the claim
2. Yes
3. No

If the basis for the claim (3) is not correctly identified, we will consider the assessment of the reliability of the claim (question 4 below) to be incorrect. We will still code their responses for questions 5-8 below and include.

4. Was the assessment of the reliability of the claim correct?

| If the basis is unreliable | If there was not a basis | If the basis is research | | |
| --- | --- | --- | --- | --- |
|  |  | without information about the size of the study or whether groups were randomly created | and either the size of the study or whether groups were randomly created is noted | and both the size of the study and whether groups were randomly created is noted |
| **A) Yes**, if the answer is Unreliable | **B) Yes**, if the answer is Can’t tell or Unreliable | **D) Yes**, if the answer is Can’t tell | **F) Yes**, if the answer is Can’t tell | **I) Yes**, if the answer is Reliable |
| **B) No**, if the answer is Can’t tell or Reliable | **C) No**, if the answer is Reliable | **E) No**, if the answer is Reliable or Unreliable | **G) No**, if the answer is Unreliable | **J) No**, if the answer is Unreliable or Can’t tell |
|  |  |  | **H) Partially**, if the answer is Reliable |  |

In the answer to question 6:

5. Was the reliability of the claim considered?

1. Yes
2. No

6. Were the advantages considered?

1. Yes
2. No

7. Were the disadvantages considered?

1. Yes
2. No

8. Were any of the following considered?

1. The student’s personal experience
2. What health professionals or researchers advise
